# Impacts of COVID-19 on Trade and Economic Aspects of Food Security: Evidence from 45 Developing Countries

**DOI:** 10.3390/ijerph17165775

**Published:** 2020-08-10

**Authors:** Vasilii Erokhin, Tianming Gao

**Affiliations:** School of Economics and Management, Harbin Engineering University, Harbin 150001, China; basilic@list.ru

**Keywords:** COVID-19, food inflation, food trade, food security, food self-sufficiency

## Abstract

The stability of food supply chains is crucial to the food security of people around the world. Since the beginning of 2020, this stability has been undergoing one of the most vigorous pressure tests ever due to the COVID-19 outbreak. From a mere health issue, the pandemic has turned into an economic threat to food security globally in the forms of lockdowns, economic decline, food trade restrictions, and rising food inflation. It is safe to assume that the novel health crisis has badly struck the least developed and developing economies, where people are particularly vulnerable to hunger and malnutrition. However, due to the recency of the COVID-19 problem, the impacts of macroeconomic fluctuations on food insecurity have remained scantily explored. In this study, the authors attempted to bridge this gap by revealing interactions between the food security status of people and the dynamics of COVID-19 cases, food trade, food inflation, and currency volatilities. The study was performed in the cases of 45 developing economies distributed to three groups by the level of income. The consecutive application of the autoregressive distributed lag method, Yamamoto’s causality test, and variance decomposition analysis allowed the authors to find the food insecurity effects of COVID-19 to be more perceptible in upper-middle-income economies than in the least developed countries. In the latter, food security risks attributed to the emergence of the health crisis were mainly related to economic access to adequate food supply (food inflation), whereas in higher-income developing economies, availability-sided food security risks (food trade restrictions and currency depreciation) were more prevalent. The approach presented in this paper contributes to the establishment of a methodology framework that may equip decision-makers with up-to-date estimations of health crisis effects on economic parameters of food availability and access to staples in food-insecure communities.

## 1. Introduction

Over the decades, food insecurity concerns have been emerging, along with the growth of the world population. Among the United Nations Sustainable Development Goals [1] to be achieved by 2030, ending hunger and establishing food security hold an important place. However, despite the best efforts of the international community to combat food insecurity across the globe, the number of undernourished people has resumed growth in 2015, after a steady decline during the 1990–2000s. The Food and Agriculture Organization (FAO) reports that over 820 million people in the world suffer from hunger, while about two billion people experience moderate or severe food insecurity [2]. In view of the fact that an additional 60 million people have become affected by hunger since 2014, the number of undernourished people is projected to exceed 840 million by 2030 [3]. In the past, the main reasons for food shortages used to be droughts and other natural disasters. With the emergence of globalization, food security has become an economic rather than an agricultural issue. Being a combination of the physical availability of food and economic access to adequate supply [4], food security at the national level is increasingly influenced by international exchange, foreign trade policies, and macroeconomic parameters of the global food market.

Most recently, the global food supply system has encountered one of the most vigorous pressure tests ever due to the outbreak of COVID-19. Vos et al. [5] emphasize the difference between the new pandemic and many of the previous ones. While, for example, Severe Acute Respiratory Syndrome (SARS), Middle East Respiratory Syndrome (MERS), and avian flu led to food shortages in affected areas causing direct damage to livestock sectors, the COVID-19 outbreak in just a few months has emerged into one of the greatest global health challenges. By the end of June 2020, 188 countries had reported cases of COVID-19 [6]. In the first two quarters of 2020, more than 11 million cases of infection and more than 530 thousand deaths had been confirmed [6]. The outbreak has turned out to be an economic challenge. While in past years, the FAO recognized military conflicts and climate extremes as main threats to food security, the 2020 report emphasized that pandemic-related economic slowdowns and downturns undermined efforts to end hunger worldwide [3]. The pandemic is not only impacting people’s lives but is disrupting food supply chains [7,8,9]. As the virus spreads and public health protection measures tighten, there are many ways in which the global food system has been strained (border closures, quarantines, supply chain disruptions, etc.). Welsh [10] explains that the pandemic is affecting food systems directly by distorting supply and demand internationally, and indirectly by degrading the purchasing power of the population and by undermining the capacity to produce and distribute food. Devereux et al. [11], Bakalis et al. [12], and Farrell et al. [13] also emphasize the two-faced nature of direct (disrupted food systems) and indirect (undermined economic access to food) effects of COVID-19 on food security. According to the United Nations World Food Program (WFP) [14], the economic impact of the pandemic could result in doubling the number of people suffering acute hunger by the end of 2020. The FAO estimates that the COVID-19 pandemic may add 83–132 million people to the number of the undernourished in 2020 [3].

For several years now, global trade systems have been increasingly distorted by various protectionist measures and trade restrictions. Commonly, governments seek to make their countries self-sufficient in food by protecting domestic food producers. In many developing and least developed countries (LDCs), export bans have been introduced to curb food inflation and establish reserves of staple foods. The World Trade Organization (WTO) [15] reports that since the start of the pandemic, Kyrgyzstan, North Macedonia, Ukraine, Thailand, and Egypt notified export prohibitions on various food and agricultural products and specifically attributed those measures to COVID-19. Russia, the world’s largest wheat exporter, and Vietnam, the third-largest rice exporter, introduced temporary export-restrictive measures. The pandemic has aggravated US–China trade tensions, in which tariffs on food trade have been intensively used as a tool of economic pressure. Most countries have implemented higher customs controls on cargo vessels, with a particular risk to perishable foods and with the risk of jeopardizing shipping activities [16]. There have been policy measures aimed at avoiding the spread of COVID-19 that might slow down food production and increase market prices globally (for instance, more stringent health standards in farms and food factories, restrictions on the movement of seasonal agricultural workers, shortage of fertilizers, veterinary medicines, and other input) [16]. In total, at least two-thirds of countries have put in place a variety of protectionist measures [15] that currently affect around 5% of globally traded food and agricultural products [17].

As governments across the world impose food export restrictions and lockdowns, such responses to the emerging health threat could create extreme volatilities in the market, provoking food shortages and resulting in food crises. For instance, during the 2007–2008 global economic downturn, the doubling of world food prices was mainly attributed to trade restrictions imposed by the largest exporters of rice, wheat, and soybeans [18,19,20,21]. The actions aimed to avert national shortages in some countries contributed to breaking the logistics chains for staple foods in many national markets [22]. Developing countries are particularly vulnerable to such distortions. In 2020, the WFP estimates the most substantial increase in the number of people suffering acute hunger to happen across LDCs in Sub-Saharan Africa and developing economies of the Middle East and Latin America [14]. Most of these countries are net importers of food and agricultural products. According to the FAO [2], out of 65 developing economies and LDCs where recent adverse impacts of the economic downturn due to the COVID-19 pandemic on food security have been strongest, 52 countries rely heavily on agricultural imports. In a situation of disrupting food supply, dependence on imports tremendously threatens the food security of those nations, especially when export restrictions are imposed by the world’s leading suppliers like Russia, Vietnam, and Ukraine. As the spread of COVID-19 and strict quarantine measures trigger economic decline, even developed economies experience food price rises unless the governments take preventive actions or retailers absorb some of the costs. Since February 2020, the global average price for rice has increased by 7.1%, for meat of cattle—by 7.0%, for meat of chicken—by 5.5%, for potatoes—by 8.3% [23]. Due to a limited capacity to produce staple crops domestically, developing economies are more vulnerable to food inflation and supply shortages. In just the first three months of 2020 at the very onset of the COVID-19 outbreak, over fifteen developing countries already experienced an increase in the cost of a basket of food staples (over 10%) [24]. At many markets, food prices have been increasing because of local logistical problems [16]. There is also a dependence of developing countries with their limited resources on a small range of food products exported to a few markets [25], many of which have been affected by the COVID-19 outbreak [15]. In one of the trade scenarios simulated by Vos et al. [5], a 1% global economic slowdown due to the pandemic could cause a decline in developing-country agri-food exports by almost 25%.

There is an array of studies that address trade aspects of food supply [26,27,28,29,30], but the majority of them focus on food self-sufficiency rather than food security. According to the FAO [31], a self-sufficient country satisfies its needs in food by means of domestic production. Although some developing countries of Africa (Mali and Senegal) and Latin America (Bolivia, Ecuador, and Venezuela) have embraced the idea of food self-sufficiency in their national policies [32], progressing liberalization of food trade over the past few decades has refocused attention from self-sufficiency, a concept that is often related to protectionism and even autarky [33], to food security. The latter incorporates a wider range of parameters of food availability, economic access to adequate nutrition, utilization of nutrients, and stability of food supply [4]. While Wegren and Elvestad [34], Meskhia [35], Clapp [36], and Saidi and Diouri [37], among others, argue that food security is about establishing a balance between domestic production and imports, many studies categorize food security as agricultural [38,39], economic [40,41], or health [42,43,44] issues rather than as a trade one.

However, at the height of the COVID-19 crisis, both larger demand gaps and higher food price surges suggest that international trade policies play a more pervasive role in ensuring food security at the national level than previously thought. Against the backdrop of the health crisis, an increase in the number of undernourished people is coupled with a global economic slowdown [2]—a trilemma that has not been adequately explored in previous studies. Lockdowns, export restrictions, and quarantine measures exacerbate these problems and call for the investigation of trade and economic impacts on food security in a new reality of food chain disruptions. So far, empirical assessments of the pandemic’s effects on food and agriculture sectors have been grouped around international organizations, such as FAO (Food and Agriculture Organization of the United Nations), WHO (World Health Organization), WFP (World Food Programme), UNCTAD (United Nations Commission on Trade and Development), and IFPRI (International Food Policy Research Institute). In a collective study compiled by the UNCTAD’s Committee for the Coordination of Statistical Activities [45], three dozen organizations and institutions identified the pandemic’s primary channels of transmission to food and agriculture sectors, and quantified the potential impacts of COVID-19 outbreak on agricultural input markets, food trade, and food consumption. Since the report covered a wide range of topics, it thus was not particularly focused on the analysis of trade-related aspects of food security. In the regional section of the report, the economic and trade impacts of COVID-19 on food security were not detailed for developing economies and LDCs. In the IFPRI study that particularly focused on developing countries, Vos et al. [5] applied a general equilibrium model to assess possible impacts of the pandemic on prices, income, and poverty. The impacts of productivity declines on prices of some food products were forecasted, as well as on income of households, but the parameters of food availability and access to food and agricultural products were not addressed.

Similarly, trade impacts on food supply chains have remained scantily explored across the array of studies on COVID-19 that have emerged in 2020. Most of these recent papers have particularly emphasized the aspects of food safety [46], agriculture productivity [47,48], and healthy nutrition [49], rather than of food imports and trade balance as dimensions of food availability. The impacts of both international exchange fluctuations and food inflation on the access pillar of food security have also remained under investigated. The link between price increase and access to food has been primarily considered in terms of supply disruptions and shortages along the retail food supply chain [50,51], not currency exchange rates. To the best of the authors’ knowledge, there are no comprehensive studies that link the incidence rates of COVID-19 with either the number of undernourished people or the degree of dependency on food imports.

In this study, the authors attempt to bridge the said gaps in the “COVID-19–trade–food security” agenda by (1) identifying interactions between the number of COVID-19 cases on one side and availability and access pillars of food security on the other; (2) assessing the cointegration between the pandemic-induced trade parameters of food availability and the number of undernourished people; (3) revealing the impacts of food inflation and currency exchange volatilities amid the global health crisis on economic access to food in domestic markets; and (4) detailing the analysis of above-mentioned health, food security, trade, and economic parameters across an array of developing economies and LDCs.

## 2. Materials and Methods

### 2.1. Variables and Data

The approach employed in this study is to assess the relationships between the food security parameter, on one side, and health and macroeconomic variables that affect availability and access pillars of food security, on the other. The number of people with insufficient food consumption (*Y*) was used as a dependent variable (Table 1). It is defined by the World Food Programme (WFP) [52] as a total number of individuals with a poor or borderline level of food consumption. Poor food consumption refers to households that are “not consuming staples and vegetables every day and never or very seldom consume protein-rich food such as meat and dairy” [52]. Households in borderline food consumption status are those “consuming staples and vegetables every day, accompanied by oil and pulses a few times a week” [52].

The number of confirmed COVID-19 cases (*X*_1_) was utilized to show the overall effect of the pandemic on food security. The selection of *X*_2–4_ variables is based on the WFP’s Hunger Map patterns. Balance of food trade (*X*_2_) reflected the dependency of a country on food imports and thus demonstrated the changes in the food supply. Food inflation (*X*_3_) and currency exchange (*X*_4_) were used to reveal the influence of changes in access to food and agricultural products on food security. The choice well correlates with the recommendations of Huseynov [53], who used exchange rate, inflation rate, and food trade as variables to identify short-term and long-term effects on food security.

The period of analysis included six months from January 2020 (when first COVID-19 cases were confirmed outside China) till the end of June 2020 (when many countries gradually lowered health alert levels). The data were obtained from the WFP’s Hunger Map portal [52], Trading Economics [54], and the United Nations Conference on Trade and Development (UNCTAD) [45,55].

### 2.2. Countries

Among developing economies, the WFP [52] reports 93 countries that experience the prevalence of undernourishment (PoU), a percentage of people in the total population that are in the condition in which an individual’s habitual food consumption is insufficient to provide the amount of dietary energy required to maintain a normal, active, healthy life. The study proceeded with 45 economies where the PoU was the highest as of 30 June 2020 (Table 2). To reveal diverse effects on food security across a variety of macroeconomic environments, the selected countries were distributed into three groups. Group 1 included low-income economies defined by the World Bank [56] as those with a gross national income (GNI) per capita of $1025 or less. Group 2 comprised lower-middle-income economies with a GNI per capita between $1026 and $3995. In Group 3, we included countries with the upper-middle level of GNI per capita between $3996 and $12,375.

**Figure 1:**
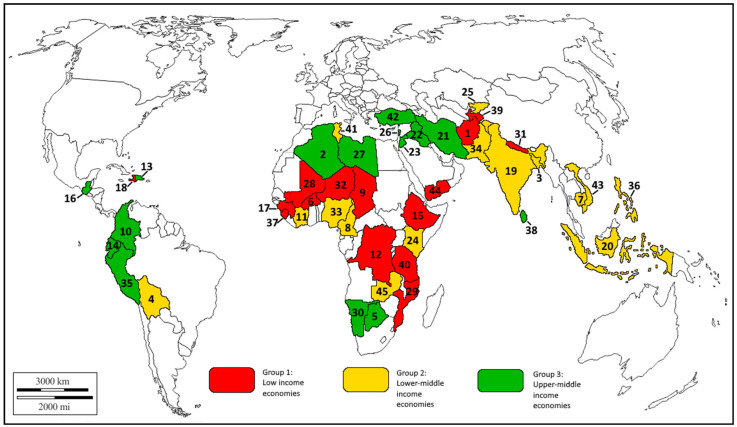
Countries included in the study. Note: 1 = Afghanistan; 2 = Algeria; 3 = Bangladesh; 4 = Bolivia; 5 = Botswana; 6 = Burkina Faso; 7 = Cambodia; 8 = Cameroon; 9 = Chad; 10 = Colombia; 11 = Cote d’Ivoire; 12 = Democratic Republic of the Congo; 13 = Dominican Republic; 14 = Ecuador; 15 = Ethiopia; 16 = Guatemala; 17 = Guinea; 18 = Haiti; 19 = India; 20 = Indonesia; 21 = Iran; 22 = Iraq; 23 = Jordan; 24 = Kenya; 25 = Kyrgyzstan; 26 = Lebanon; 27 = Libya; 28 = Mali; 29 = Mozambique; 30 = Namibia; 31 = Nepal; 32 = Niger; 33 = Nigeria; 34 = Pakistan; 35 = Peru; 36 = Philippines; 37 = Sierra Leone; 38 = Sri Lanka; 39 = Tajikistan; 40 = Tanzania; 41 = Tunisia; 42 = Turkey; 43 = Vietnam; 44 = Yemen; 45 = Zambia. Source: Authors’ development.

To capture potential divergences in both economic and geographical specificities of food security, we selected the countries from six regions of Africa, Asia, Latin America, and the Middle East (Figure 1). The classification by income group is based on the World Bank Atlas method [57], while that by geographic region—on the World Bank country classification [56].

### 2.3. Study Hypotheses

The following hypotheses were established to reflect supposed variations in *Y–X_(1–4)_* interactions depending on the level of income:

The COVID-19 pandemic has hurt the entire international community ranging from the least developed countries of Africa to the wealthiest economies of Europe and America [16,58]. Therefore, we hypothesize that:

**Hypothesis** **1.** *X_1_ exert a direct strong effect on Y across Groups 1–3 countries without regard to income level*.

In low-income economies, food shortages and other disruptions of food availability are commonly considered to be the major factors of food insecurity [59,60,61,62,63]. Additionally, due to the world’s highest portion of disposable income spent on food in low-income countries [64,65,66,67], even a slight deterioration in the economic access to staple foods harms the food security status of the households. As the spread of the COVID-19 and lockdown measures trigger economic decline, we suggest that:

**Hypothesis** **2.** *In Group 1 countries, the strongest influence over Y is exerted by food availability and food access parameters, X_2_ and X_3_, respectively*.

With a rise in income level, a portion of imported food in consumption increases due to importing higher-quality and pricier products [68,69,70], whereas the fluctuations in world prices and import volumes exert stronger effects on the food security status of the households compared to those of domestic supply. In a situation when global food supply chains are disrupted by currency exchange volatilities and trade restrictions amid the COVID-19 pandemic, we assume that:

**Hypothesis** **3.** *In Group 3 countries, the effects of X_4_ and X_2_ on Y are the highest among the economies included in the study, while that of X_3_ is the lowest*.

### 2.4. Study Stages

Across the array of six variables, the four-stage analysis was conducted individually for each of the forty-five countries (Table 3). At Stage 1, a stationary test was conducted to check whether a co-integration existed between the selected variables. To illustrate short and long-run interactions between the variables, the autoregressive distributed lag (ARDL) method was employed at Stage 2. Then, we applied Yamamoto’s causality test to reveal the causality directions of the variables. Finally, by utilizing the variance decomposition approach, we attempted to predict the future relative strengths of causalities between the variables.

Prior to the identification of the relationships between the variables, it is important to understand if the data is stationary [71,72]. The system behaves correctly in the case where static and dynamic properties of the variables remain unchanged and values of the system state belong to an acceptable interval [73]. A variety of approaches have been developed to check the stationarity and to investigate further the cointegrating interactions between stationary variables. Concerning macroeconomic parameters, the most commonly used techniques are the ADF test by Dickey and Fuller [74] and the PP test by Phillips and Perron [75]. They were used by Herwartz and Reimer [76] to reveal the relationship between interest rates and inflation across developing economies, by Chang et al. [71,77] to investigate stationarity of GDP per capita in the OECD countries, by Ranjbar et al. [78] and Su et al. [79] to study income convergences in developing and least developed countries of Africa, and by Hoarau [80] to test the purchasing power parities for real exchange rates in Central America. In combination with the ADF and the PP tests, Aliyev et al. [81], Humbatova et al. [82], and Naseem et al. [83], among others, used the KPSS (Kwiatkowski–Phillips–Schmidt–Shin) unit root method to enhance the robustness of the stationary test results. The KPSS test has been widely used in macroeconomics and international finance (for instance, by Kuo and Mikkola [84], Gunduz [85], and Tsen [86]) to check long-run and functional time series for stationarity [87,88]. In short-run time series similar to those used in our study, the employment of the KPSS test might be misleading due to the invariance of the technique to seasonal dummies [89] and the duality of the level stationary and time trend stationary models used in the test [90]. To ensure the correct interpretation of data in the short-run, we abandoned the KPSS method and utilized the ADF and the PP techniques to check the cointegration between *Y* and *X*_1–4_.

After the stationarity of the variables is confirmed, the interactions between the variables may be verified. To identify the relationships between the variables within the two established multitudes, we employed the autoregressive distributed lag (ARDL) method elaborated by Pesaran et al. [91] (Equation (1)). Although the use of this technique in food security studies has been very rare, many scholars have employed the method to identify both short- and long-run relationships between various macroeconomic parameters in developing countries. For instance, Öztürk and Özdil [92] used ARDL to investigate the interplays between economic growth and unemployment in the OECD countries, Elian et al. [93] tested the relationship between foreign direct investment inflows and economic growth across the BRICS countries, and Appiah et al. [94] studied growth determinants in emerging economies. To the best of the authors’ knowledge, we have not been able to track previous applications of the ARDL method in studying availability or access-related parameters of food security. However, there are abundant examples of an effective utilization of the ARDL in revealing the interplays between domestic supply and imports [53,95], trade balances [53,96,97], inflation [98,99], international currency exchange [100,101], and health [102].

(1) $$\Delta Y_{t}=\delta_{0}+\overset{l}{\underset{i=1}{\sum }}\delta_{1i}\Delta Y_{t-i}+\overset{l}{\underset{i=1}{\sum }}\delta_{2}i\;\Delta X1_{t-i}+\overset{l}{\underset{i=1}{\sum }}\delta_{3}i\Delta X2_{t-i}+\overset{l}{\underset{i=1}{\sum }}\delta_{4i}\;\Delta X3_{t-i}+\overset{l}{\underset{i=1}{\sum }}\delta_{5i}\;\Delta X4_{t-i}+\varpi ECT_{t-1}+\varepsilon_{t-i}$$

where **Δ** = first difference operator; $\delta_{0}$ = constant term; $\delta_{1},\;\delta_{2},\;\delta_{3},\;\delta_{4},\;and\;\delta_{5}\;$= short-run elasticities of the variables; *i* = ARDL model lag order; $\varpi ECT_{t-1}$ = error correction term; $\varepsilon_{t}$ = error disturbance; *t* = time.

If F-statistics is larger than the upper critical bounds value [I(1)], the series are cointegrated. If it is below the lower critical bounds [I(0)], the variables are not cointegrated. As robustness tests for the ARDL, we utilized fully-modified ordinary least squares (FMOLS) and dynamic ordinary least squares (DOLS). According to Phillips and Hansen [103], testing the ARDL results by FMOLS allows one to correct the system for endogeneity and serial correlation effects. It is a non-parametric method to identify a correlation between the components of model error terms [104]. The approach was used by Narayan and Narayan [105], Abu [106], and Adebayo [97] to test the interactions between various macroeconomic variables, including trade volume, inflation, and currency depreciation. The rationale of using FMOLS in our study is that it allows receiving consistent parameters even in the small samples in the short-run. Additionally, it helps to overcome the problems of endogeneity and serial correlation and thus allows for the heterogeneity in the parameters [107,108]. DOLS has been commonly utilized in combination with FMOLS as a computationally convenient alternative to FMOLS estimators. According to Stock and Watson [109], DOLS is employed to estimate the equilibria that is corrected for potential simultaneity bias among explanatory variables. Similar to FMOLS, DOLS is applicable to small samples in the short term [110]. Its estimators obtained from least-squares estimates are unbiased and asymptotically efficient even in the presence of the endogenous problem [111]. Echoing the successful application of both FMOLS and DOLS in testing the robustness of the ARDL results by Yuzbashkandi and Sadi [104], Pasha and Ramzan [112], Priyankara [107], and Adebayo [97] we consider these two methods as efficient estimators to study serial interactions and examine potential correlations between *Y* and *X*_1–4_. Some scholars, for instance, Aliyev et al. [81], Guan et al. [113], Yue et al. [114], and Rahman et al. [115], further checked FMOLS and DOLS results by employing the canonical cointegration regression (CCR). The method is commonly implemented to remove the long-run dependencies between the cointegrating equation and stochastic regressors, which does not apply to small samples in the short-run used in our study.

The utilization of the ARDL method allows us to identify the interaction between the variables, but not the direction of causalities. To capture these directions, we employed a causality test elaborated by Toda and Yamamoto [116]. This technique has been extensively used by many scholars, including Pantamee et al. [117], Adebayo [97], and Bilgehan [118], to estimate causal relationships between domestic market parameters and exogenous factors across developing countries worldwide. Among the drawbacks of the TY causality test is the inability to predict the relative strength of causalities between the variables beyond the period under study. Sankaran et al. [119], Rana and Sharma [120,121], and Wang and Ngene [122] suggested to overcome this problem by using the Wald or modified Wald (MWald) tests, but Hayashi et al. [123] and Lemonte [124] demonstrated that, in small samples when used empirically to search for unimportant parameters, the Wald test procedure could be misleading. In furtherance of Zhang et al. [125], Mao et al. [126], Adebayo [97], and Chan et al. [127], we used variance decomposition instead of the Wald test to explore the strengths of inter-variables causal interactions and to reveal potential causality impacts. The method was applied for nine consecutive periods from July 2020 till March 2021.

## 3. Results

The results of the ADF and the PP tests across three groups of countries (see Supplementary Materials to this paper, Tables S1–S3) demonstrate that all five variables are stationary at a level of either I(0) or I(1). In all cases, the calculated F-statistics values exceed the upper bound (Table 4). It means that the precondition for co-integration between *Y* and *X*_1–4_ is established in all countries included in the study. The stationarity of the data series along with the revealed co-integration between the variables both confirm the appropriateness of the established data set for the ARDL analysis.

Since the study includes six periods (months), it is mainly centered on explaining the short-run relationship between the number of people with insufficient food consumption and independent variables. The ARDL short-run estimates for the three groups of countries are summarized in Table 5, the detailed per-country calculations are provided in Supplementary Materials (Tables S4–S6).

In Group 1 countries, the strongest effect on the growth of *Y* is caused by an increase in food inflation *X*_3_. This effect is statistically significant across the group. Some variables also exert strong direct influence on *Y*, for instance, *X*_2_ in Sierra Leone and Yemen and *X*_4_ in Haiti and Niger. When other factors remain constant, an increase in the number of registered COVID-19 cases by 1% results in the growth of *Y* by 0.14% in Tajikistan and by 0.05% in Mozambique and Sierra Leone. In Niger, a 0.18% increase in the number of people with insufficient food consumption is caused by a 5% rise in *X*_1_ (0.16% in Guinea, 0.14% in Tanzania, and 0.03% in Afghanistan).

In Group 2 countries, we see kaleidoscopic linkages between *Y* and *X*_1_. In Pakistan and India, the countries of South Asia which have been severely hit by the pandemic, an increase in COVID-19 cases by 1% results in the growth of the number of people with insufficient food consumption by 0.56% and 0.53%, respectively. In East Asia, on the contrary, we see that the food security status of the households improves when the number of registered COVID-19 cases goes up (when other variables remain constant). In Cambodia and Vietnam, where the growth of **∆***X*_1_ in January–June 2020 was more moderate compared to some of their neighbors in South Asia, we see a negative *X*_1_-to-*Y* relationship. The lower portion of imports in the balance of food trade has a positive and statistically significant impact on the number of people with insufficient food consumption in Cameroon, Kenya, Tunisia, and India, whereas, in Cambodia, the relationship between *X*_2_ and *Y* is negative. The effects of *X*_3_ and *X*_4_ on *Y* are positive in all countries, except Cambodia, but not that significant compared to *X*_1_ and *X*_2_.

While the increase in the number of confirmed COVID-19 cases is found to have a significant positive effect on *Y* in the countries of Latin America and the Caribbean, in the case of many other Group 3 countries, there is a negative relationship between these variables (Botswana, Namibia, Libya, Jordan, and Iran). The strongest impact of *X*_1_ on *Y* is revealed for Peru and Ecuador, where an increase in COVID-19 cases by 1% is associated with the growth of the number of people with insufficient food consumption by 0.54% and 0.40%, respectively. Statistically strong interplays are reveled between *Y* and *X*_2_ in Algeria, Botswana, and Colombia, between *Y* and *X*_3_ in Sri Lanka and Turkey, and between *Y* and *X*_4_ in Ecuador and Namibia. The negative influence of *X*_2_ on *Y* is identified to be statistically significant in Sri Lanka and Iran, of *X*_3_ on *Y*—in Algeria, Dominican Republic, Iran, and Iraq, of *X*_4_ on *Y*—in Sri Lanka. Error correction measure is statistically significant in the case of all three groups of countries.

The results of the fully-modified ordinary least squares (FMOLS) and dynamic ordinary least squares (DOLS) tests are employed to check the robustness of the ARDL estimates (Table 6 for a group-based summary, Tables S7–S9 for country-specific data).

The number of registered COVID-19 cases is confirmed to result in higher food insecurity across three types of economies included in the study, except some countries of Sub-Saharan Africa (Burkina Faso, Chad, Ethiopia, Zambia, Botswana, and Namibia), Middle East and North Africa (Yemen, Iran, Jordan, and Libya), and East Asia and Pacific (Cambodia and Vietnam). Among these twelve countries, for which we see a reverse relationship between COVID-19 cases and the number of people with insufficient food consumption, there are representatives of various income groups. Reasoning from this fact, we can assume that in a particular country, the direction of the *Y*-*X*_1_ link does not depend on GNI per capita. However, when the relationship between these two parameters is positive, there is evidence of a stronger *Y*-*X*_1_ correlation in Group 3 countries compared to that in Group 1 low-income economies. The Toda–Yamamoto test demonstrates the most significant causality flowing from the number of COVID-19 cases to the number of people with insufficient food consumption in Group 3 countries of Latin America (Colombia, Ecuador, and Peru) and Europe (Turkey), whereas, in low-income economies, the *X*_1_→*Y* causality is weaker (Table 7, Tables S10–S12).

Similarly to bidirectional interactions between COVID-19 cases and food insecurity across all groups of countries, both the FMOLS and DOLS tests confirm divergent relationships between the number of people with insufficient food consumption and the balance of food trade. In Group 2 and Group 3, *Y*-*X*_2_ relations are positive (except for Cambodia, Iran, and Sri Lanka), while in Group 1, they are negative for almost half of the countries. In Sub-Saharan Africa (Burkina Faso, Chad, Mali, Niger, and Tanzania), an increase in the balance of food trade is identified to be effective at reducing the number of people with insufficient food consumption. From the estimation of the Toda–Yamamoto causality test (Table 7), we see the unidirectional *X*_2_→*Y* causality in Group 1 countries, but the significance of the link is low.

The strongest influence of food access on food security is revealed in low-income economies of Sub-Saharan Africa (Burkina Faso, Ethiopia, Guinea, Mali, Mozambique, and Sierra Leone), as well as some countries of Central Asia (Tajikistan) and Middle East (Yemen). In some Group 3 countries, robustness tests show a negative relationship between *X*_3_ and *Y* when the number of people with insufficient food consumption goes up amid food deflation. We also see examples of such reversal links in upper-middle-income countries of the Middle East (Iran and Iraq), where food prices are to a large extent under government control. Confronting Hypothesis 3, in lower-middle-income economies of Southeast Asia (Cambodia, India, Indonesia, Pakistan), seasonal retreat in food prices does not immediately result in higher food security expectations among people. In these countries, the *X*_3_→*Y* causality link is weak due to the high portion of locally produced seasonal food in consumption. Among Group 2 economies, more significant causality flowing from food inflation to food insecurity is revealed for the countries of Sub-Saharan Africa (Cote d’Ivoire, Nigeria, Zambia, Kenya), where diversity of locally-produced staples is narrower compared to Asia. When a portion of marketed food in supply is higher, a deterioration in economic access to marketed products imposes a more significant impact on the aggravation of food insecurity.

It is assumed that in the countries where a large portion of the food supply is ensured by imports, food inflation might correlate with currency exchange. However, we see that in low-income economies, where food access strongly correlates with food inflation, the number of people with insufficient food consumption is marginally affected by currency exchange fluctuations. The weaker link between *Y* and *X*_4_ across Group 1 stems from the fact that low-income economies import a considerably lower amount of high-quality and expensive food products compared to lower-middle and upper-middle-income countries. As contrasted with low-income countries, Group 3 economies are deeper integrated into global supply chains of value-added food products. From this perspective, amid the COVID-19 pandemic, the most significant causal relationships between volatilities in currency exchange and food supply are found in the countries with the highest GNI per capita among those included in the study—Turkey, Colombia, and Peru.

With the current dynamics of registered COVID-19 cases across three groups of countries, the extrapolation of the short-run ARDL estimates to the future forecasts a gradual increase in the proportion of food insecurity variance explained by the effects of the pandemic. Variance decomposition of *Y-X_(1–4)_* interactions (Table 8, Tables S13–S15) indicates a diversity of potential causality impacts of COVID-19 cases, balance of food trade, food inflation, and currency exchange on the number of people with insufficient food consumption.

For Group 1 countries, the decomposition analysis suggests a rather stable and weak *Y-X_1_* linkage over a three-quarters horizon (Table S13). Only in Nepal, Yemen, and Mali, the food security situation could be significantly predicted by the variations of *X*_1_. But even in these countries, we see that the expected proportions of *X*_4_ and *X*_3_ in *Y* nearly equal that of *X*_1_ in size. For most of the low-income economies, variance decomposition projects an increase in the proportion of *Y* explained by food inflation (14.84% in Ethiopia, 13.70% in Chad, 11.12% in the Democratic Republic of the Congo) and currency exchange (9.01% in Burkina Faso, 7.72% in Mali, 6.65% in Niger).

In lower-middle-income economies, the number of people with insufficient food consumption seems to be increasingly affected by food availability. By March 2021, in import-dependent Kenya and Kyrgyzstan, the proportion of *Y* explained by the balance in food trade is forecasted to exceed 10% (Table S14). The weight of food access in establishing food security will grow in the countries of East Asia (15.98% and 15.27% of *Y* explained by *X*_3_ in Vietnam and Cambodia, respectively) and Sub-Saharan Africa (12.44% in Cote d’Ivoire and 11.60% in Zambia). The projected causality between *Y* and *X*_1_ is the strongest in the countries of South Asia. In India, at the current rate of registered COVID-19 cases, almost 14.50% of the proportion of insufficient food consumption will be impacted by the pandemic. It is the highest expected impact of the pandemic on food security among forty-five countries included in the study. In Bangladesh, the strength of the *Y-X_1_* linkage will exceed 12.00% by the second quarter of 2021. Across Sub-Saharan Africa and East Asia, a relatively low number of registered COVID-19 cases allows one to predict the moderate role of *X*_1_ in the explanation of *Y* variations over a nine-month horizon.

Among upper-middle-income countries, the impact of the pandemic on the number of people with insufficient food consumption is not expected to vary significantly from region to region. The proportion of *Y* explained by *X*_1_ is expected to peak in the countries, where the number of COVID-19 cases per capita in January–June 2020 was the highest among Group 3 economies. Over the entire time horizon considered in this study, the growth in *X*_1_ will most likely and consistently be converted into a higher percentage of the population in food insecurity status in Peru, Iran, and Turkey (Table S15). Variance decomposition also projects significant contributions of *X*_1_ to *Y* in Colombia (8.93%), Algeria (8.35%), and Ecuador (6.91%).

## 4. Discussion

The revealed interplays between the variables across three groups of countries allowed us to test the hypotheses:

**Hypothesis** **1:** **Not confirmed**. *The X_1_-Y relationship is uneven across Group 1–3 countries, where the strength of causal interaction between the two variables increases with the growth of income level*.

The effect of the COVID-19 outbreak on the number of people with insufficient food consumption is observed across the three groups of countries. This finding supports the expectations of the FAO [3,16], the WFP [14], and the WTO [15], as well as the projections of many scholars [5,10,50], who say that the spread of COVID-19 may bring damage to global food security, particularly painful in the least developed and developing economies. According to our results, the number of registered COVID-19 cases is indeed associated with higher food insecurity in many countries included in the study. The *Y*-*X*_1_ linkage is statistically significant in the countries (primarily, middle-income economies) where the number of registered COVID-19 cases per capita is high (Pakistan, India, Peru, Ecuador, Turkey).

Across low-income economies; however, the impact of COVID-19 on food insecurity is much weaker compared to that in upper-middle-income countries. This result well agrees with the FAO’s estimation that higher-income countries are more likely to face food supply disruptions during the novel health crisis, given their deeper integration in global supply chains and capital-intensive agricultural systems [45]. In 2019, the WFP [14] reported Yemen, the Democratic Republic of Congo, Afghanistan, Venezuela, Ethiopia, South Sudan, Syria, Sudan, Nigeria, and Haiti to constitute the worst food crises. Confronting the established Hypothesis 1, we see that in most of these countries, the relationship between the number of people with insufficient food consumption and the number of COVID-19 cases is not strong but moderate. For example, in Afghanistan, where at least 35% of the population is in a state of food crisis [14], the increase in the number of COVID-19 cases by 5% results in the growth in food insecurity by only 0.03%. Moreover, we see that in several low-income countries, the dynamics of COVID-19 cases is related to *Y* in a negative way. In some countries, where the number of COVID-19 cases remained low during January–June 2020, there is a reversal *Y*-*X*_1_ relationship. In Haiti, an increase in the number of COVID-19 cases by 1% is associated with the improvement in the food security status of the population by 0.11% (by 0.03% in Nepal and by 0.02% in Chad). Such a relationship can be explained by a statistically insignificant correlation between *X*_1_ and *Y* due to the low number of confirmed COVID-19 cases per capita.

Still, the effects of the pandemic on food security in low-income countries should not be underestimated. Even without considering the direct health-related influences of the spreading COVID-19 virus, the FAO projects low-income economies of Africa to overtake both lower-middle-income and upper-middle-income countries of Asia and Latin America to become the region with the highest number of undernourished people in 2030 [3]. COVID-19 could exacerbate this trend, while the effects of the current health crisis on food security may be amplified by local outbreaks of other diseases that have been endemic in Africa and Asia. Many scholars, including Mouloudj et al. [128], Bakalis et al. [12], Poudel et al. [129], and Siche [130], witnessed significant adverse effects of SARS, MERS, avian and swine flu, Ebola, and other outbreaks on both agricultural production and food consumption behavior. On a smaller scale and in a more localized context, endemic diseases cause disruptions across local food supply chains similar to those the COVID-19 pandemic does to the global food supply. According to Ceylan and Ozkan [131], both SARS and MERS had a downsizing effect on the production and supply of food, as well as on labor demand in agriculture. Kodish et al. [132] and Wernery and Woo [133] found movement restriction policies and quarantines introduced during MERS, Ebola, and other more local outbreaks to have substantial effects on agricultural production, food industry, as well as on distribution and retailing of many staples. Dounamou et al. [134] revealed a significant shift in consumption patterns during Ebola outbreaks in West Africa. In an attempt to avoid the consumption of wild meat potentially associated with the Ebola virus disease, many people tend to switch to domestic meat and fish. In a situation when affordability and availability of alternative protein sources are deteriorated by trade and economic factors (as we see it amid the COVID-19 pandemic), local outbreaks of other diseases may substantially aggravate both health and food security status of broad segments of the population.

Transmissibility of COVID-19 is estimated to be 2.5 compared with 2.4 for SARS. Other recent pandemics had lower basic reproductive rates—1.5 for the 2009 influenza pandemic and only 0.9 for MERS [135]. Despite comparable transmissibility rates, the trajectories of COVID-19 and SARS are different. While SARS 2003 outbreak was contained within eight months with a global total of 8098 reported cases across 26 countries [136] and MERS caused 2494 reported cases in 27 countries [137], COVID-19 is spreading rapidly with over 10 million known cases as the end of June 2020. But the unprecedented spread of COVID-19 throughout the world compared with other pandemics of the past is caused by greater ease of global transportation [138] and higher population density [12] the world has achieved by 2020, not exclusively by higher contagiousness or better transmissibility of the novel coronavirus. With the growing globalization, any local outbreak has its chance to emerge to the global pandemic, while climate change and environmental degradation may increase the appearance of zoonotic diseases in humans [139,140]. In LDCs and developing countries of Africa, Asia, and the Middle East, the impact of outbreaks on the food security status of people is particularly severe in transitional food value chains, such as wet markets [141]. They bring together large numbers of people in crowded spaces at considerable risk of contagion [142]. According to Hasöksüz et al. [143] and Silva-Jaimes [144], in such traditional food markets where human–wildlife interactions and cross-species infections are frequent, novel coronaviruses are likely to emerge periodically. Petersen et al. [135] also expect a post-COVID-19 pandemic of another coronavirus, an influenza virus, a paramyxovirus, or a completely new disease to be highly likely in the nearest future. Due to rather high economic and social costs of bringing local outbreaks under a successful level of control at early stages [145], LDCs and developing countries of Africa and South Asia are particularly vulnerable to the frequency and intensity of disease cycles that may realize their “pandemic potential”.

On top of the health and economic effects of COVID-19, there are climatic pressures that often aggravate supply-side food shocks in Africa and Asia (droughts, heatwaves, locust swarms, etc.) [7]. In 2020, production declines due to dry weather conditions are expected in Morocco and Tunisia [146]. In East Africa and South Asia, significant rainfall amounts resulted in floods and caused damages to farmland and livestock deaths. Zurayk [50] has recognized locust invasion in the countries of the Middle East and East Africa as a further destabilizer of the stability of food supply in the times of the pandemic. Shilomboleni [147] prognoses the COVID-19 pandemic to put a further strain on Africa’s agricultural sector amid the recent desert locust outbreak in the Horn of Africa. In West Africa, COVID-19 lockdowns are limiting population movement and causing local labor supply shortages [146]. According to FAO’s crop prospects [146], adverse weather resulted in a below-average output in North Africa and Central Asia and near-average cereal harvests in Central America and the Caribbean. Amid such climate-change driven disruptions of food systems, the pressure of both COVID-19 and local outbreaks on food consumption may be intensified by lower harvests and higher food prices in Group 1 countries, as well as across a wider community of developing economies. Mouloudj et al. [128] and Janssens et al. [148] expect developing countries of Africa and Asia in which agriculture contributes significantly to GDP (Sierra Leone, Chad, Niger, Mali, Cambodia, and Vietnam) to be affected by both climate and economic effects of the pandemic (suspension of agricultural activities, agricultural labor lockdowns, etc.). According to the FAO estimates [146], over 14 million people in Africa in 2020 need urgent food assistance, including 7 million in Nigeria, 2.1 million in Burkina Faso, 2 million in Niger, 1.3 million in Mali and Sierra Leone, and 1 million in Chad. With respect to food availability, domestic agricultural production in LDCs and developing countries of Africa may be severely affected by the disruption of the supply of various inputs [13], including animal feed and ingredients for food product preparation, especially if they need to be imported [145].

**Hypothesis** **2:** **Partly confirmed.***In Group 1 economies, the influence of food inflation over access to food and agricultural products is stronger than that of food trade over food availability*.

In low-income economies, the food security status of people is significantly influenced by both the physical availability of and economic access to food products. According to FAO’s most recent food security report, a key reason of growing food insecurity in developing countries is that many people cannot afford the increasing cost of healthy diets, while the nutritional status of vulnerable population groups has been deteriorated due to the economic impacts of COVID-19 [3]. Martin and Anderson [20] and Freund and Özden [149] assumed that protectionist trade policy could bring a risk of additional economic losses for developing countries by insulating domestic markets from global food price fluctuations. The FAO’s monitoring of food price changes [23] since February 2020 demonstrates that amid the COVID-19 crisis, trade restrictions are imposed against the backdrop of growing food prices. The FAO Food Price Index averaged 93.2 points in June 2020 (by 2.4% higher than in May 2020) [150]. Zurayk [50] reports a global price increase in the food basket of 20% to 50% with the prices of dairy products, vegetable oils, sugar, and other food and agricultural products rebounded to multi-month highs [150]. Our results indicate that rising food inflation deteriorates food access across Group 1 countries as it is tightly linked with the increasing number of people with insufficient food consumption. This correlates with FAO’s estimation that the cost of a healthy diet in 2020 has exceeded the international poverty line, making it unaffordable for the poor and thus fueling food insecurity in most developing countries, particularly in Sub-Saharan Africa and Southern Asia [3]. Healthy diets have become 60% less affordable compared to the nutrient adequate diets and five times more expensive than diets that meet only dietary energy needs through a starchy staple [3]. Many scholars, including Bakalis et al. [12], Berkowitz et al. [151], Gundersen and Ziliak [152], and Garcia et al. [153], associate undernourishment with adverse health outcomes, including chronic conditions, mental health challenges, and increased risk of mortality. Niles et al. [154] found that lower economic access to food forced many food-insecure households to disrupt eating, cut meals, eat less to stretch their food, or even go hungry. This link between the cost of a diet and food security status has an important impact on individual health. An increase in food inflation is confirmed to have a significant effect on food insecurity in Group 1 countries, thereby supporting Hypothesis 2 and confirming previous findings of Smith et al. [59], Power [66], Sonnino et al. [67], Esturk and Ören [155], and many other authors who linked food insecurity with the level of income rather than with food imports. In our study, the strongest influence of food inflation on the number of people with insufficient food consumption is revealed in low-income economies of Sub-Saharan Africa (Burkina Faso, Ethiopia, Guinea, among others), as well as some countries of Asia and the Middle East. The UNCTAD [45] also acknowledged the countries of Sub-Saharan Africa to be particularly exposed to demand-side risks of food access during the COVID-19 crisis, including contracting incomes, downturns in economic growth, undernutrition, and micronutrient deficiencies in response to income shocks.

Food inflation affects demand, but inflation itself is often a product of changing demand patterns. During the economic crisis of 2008–2009, reduced income made people spend less and resulted in shrinking demand for food [16]. The novel health crisis is quite a different story. On the back of rising lockdown fears in February–March 2020, food inflation was fueled by higher demand due to panic buying [145,156]. Although Yuen et al. [157], Zurayk [50], and Fawzi et al. [156] did not account for the level of income as a factor that affected such consumer behavior, we may assume the contribution of panic buying to food inflation to be more significant in Group 3 countries. In low and lower-middle-income economies, people have less free money to stock up food, while most cases of panic buying have been evidenced in developed countries [158,159]. In LDCs and developing economies, no significant spikes in food demand have been registered in the first quarter of 2020. On the contrary, the FAO [16] expects the crisis-induced economic downturn to alter dietary patterns in the developing world due to a disproportionately larger decline in consumption of higher-value products like meat, fish, fruits, and vegetables.

Many scholars [160,161,162] have found the likelihood of food insecurity to increase with income inequality. According to the FAO [2], the inequality–insecurity link is 20% stronger for low-income economies compared with middle-income ones. This well agrees with our finding of the disproportional effects of food inflation on food insecurity across the three groups of countries. For instance, in Mozambique (Group 1), keeping other variables constant, a 1% increase in food inflation leads to a growth in the number of people in food insecurity status by 0.80% (by 0.71% in Tajikistan, by 0.53% in Burkina Faso, 0.41% in Guinea, and so on down the list of Group 1 economies). In Group 2, the *X*_3_-*Y* link is weaker while that in Group 3 is the weakest among the countries included in the study. There are even negative relationships between *X*_3_ and *Y* in some Group 3 countries of the Middle East and Southeast Asia.

With that said, our study demonstrates that in lower-middle and upper-middle-income developing countries, the causality link between food inflation and food security is weaker compared with that in LDCs. Generally, in low-income countries, food supply is for the most part ensured by local staple foods, whereas extensive import is prohibitively expensive. According to the FAO [3], low-income countries rely more on staple foods and less on fruits and vegetables and animal source foods than high-income countries. As previously found by Thome et al. [65], Ritchie et al. [62], and Elbushra and Ahmed [163], weak cointegration between food inflation and food security in low-income economies could be explained by the high portion of locally produced staples in consumption. Amid the COVID-19 outbreak, some countries have decreased food purchases from abroad, thus automatically increasing their foreign trade balances due to the lower portion of imports. As more households switched to locally produced staples, their food security status improved. However, as noticed by Devereux et al. [11] and Farrell et al. [13], a closure of open-air markets and a ban on street vendors (the two most common food outlets in poorer countries) may disrupt food access even in a situation when consumption is reoriented on local products. Prior to the current health crisis, many food-insecure households have reported such food coping strategies as, for example, seeking resources from the charitable food sector or relying on social networks for support [164,165]. Amidst COVID-19 lockdowns and restrictions, most of the nutrition assistance programs have been frozen.

Therefore, it is revealed that food availability seems to be strongly related to the food security status of households, but through local supply, not import. Following Deuss [166], Martin and Anderson [20], and Hendrix [19], we assume that food trade restrictions were more pronounced in the countries with a higher import dependency. According to Wood et al. [167], for import-dependent economies, both global food chain disruptions and protectionist trade policies on the part of key suppliers could have serious negative consequences for food security. This agrees with Puma et al. [21], who found that LDCs suffer greater import losses due to disruption of food supply chains through their increased dependence on imports of staple foods. There is a unidirectional *X*_2_→*Y* causality across Group 1, but the significance of the link is low even in the countries where food availability largely depends on imports (Haiti, Guinea, Tajikistan). These findings do not support Hypothesis 2. With an increase in the level of income, the link between food trade balance and food availability becomes tighter. The strongest effect of *X*_2_ on *Y* is revealed for import-dependent upper-middle-income economies (Jordan, Lebanon, Botswana, Algeria, Colombia). In most low-income countries, we see how a lower proportion of food imports in trade amid the COVID-19 outbreak is associated with a reduction in the number of people with insufficient food consumption.

**Hypothesis** **3:** **Confirmed.***Different from the low-income economies, in Group 3 countries, the food security status of people is affected by food trade and currency exchange rather than by food inflation*.

As recognized by Wood et al. [167] and Hendrix [19], food import is particularly essential to LDCs for meeting the dietary needs of their population during the COVID-19 outbreak. Our results; however, suggest that Group 1 and Group 2 economies rely on less diversified imports compared to Group 3 countries which are deeper integrated into global supply chains. For the latter, higher dependence on imports results in a stronger influence of food trade balance and currency exchange on food supply and, consequently, on the food security status of people. While Devereux et al. [11] stated that COVID-19 had not compromised food supply globally, Mouloudj et al. [128] and Toffolutti et al. [8] found food security status of developing countries that depended on imports of staples to be particularly threatened by disruptions of the food supply in the first half of 2020. In import-dependent developing countries, currency depreciation drives up the cost of food imports [49]. Thus amid market uncertainties induced by the COVID-19 crisis, currency exchange becomes a factor of both food availability (more expensive imports due to currency depreciation) and access to food (the higher price of imported food on the domestic market when expressed in national currency). The UNCTAD [45] revealed heightened risks to food security in those countries of North Africa and the Middle East that rely on food imports and thus are dependent on currency volatilities triggered by the pandemic. In support of this UNCTAD’s estimation, the strongest effects of *X*_2_ and *X*_4_ on *Y* are found for Algeria and Turkey. In furtherance of Hypothesis 3, we expect an increase in the proportion of *Y* explained by both food trade and currency exchange, particularly, in upper-middle-income countries. In Libya, where the dependency on food imports exceeds 90%, the impact of *X*_2_ on *Y* is projected to be the highest among the three groups of countries (18.03%). In Namibia, another Group 3 country largely dependent on imports, the proportion of *X*_2_ in *Y* will almost reach 12.20% by March 2021. The importance of currency exchange in securing food supply will go up in the countries deeply integrated into global food supply chains. For instance, in Turkey, 15.21% of *Y* will be explained by *X*_4_.

The effect of food inflation on the number of people with insufficient food consumption is found to be weaker across upper-middle-income economies compared to that in low-income countries. This finding both supports Hypothesis 3 and agrees with Frankenberg and Thomas [168] and Smith and Glauber [69], who revealed that higher prices for staple foods aggravated poverty traps for low-income households, but might not have much effect on the food security status of relatively well-off households. On the other hand, domestic price volatility may be exacerbated by trade restrictions that have been implemented by some Group 2 and Group 3 countries on the backdrop of the COVID-19 outbreak. In the studies on the effects of export restrictions during the global crisis of 2007–2008, Deuss [166] and Djuric et al. [169] demonstrated that protectionist policies did not achieve their objective of reducing price volatility in the country imposing the restriction. There are also studies that show how trade restrictions resulted in food price spikes during the food crises in 1973–1974 [170], 1986–1988 [171], 2006–2008 [21,172,173,174], and 2010–2011 [20]. Dawe and Timmer [175] and Abbott [176] found that, in the short-run, an export ban could be a successful decision to ensure the food security of a country by both establishing a reserve of staples and isolating domestic market from the global price volatility. For instance, in Cambodia, that limited exports of certain agricultural products in March–April 2020, we see how both negative balances of food trade and low food inflation resulted in the reduction in the number of people with insufficient food consumption. For Vietnam and Turkey, on the contrary, their decisions to restrict food export have not brought much success. The ARDL analysis demonstrates that in Vietnam, a 1% change in the food trade balance is associated with an increase in food insecurity by 0.02%. In Turkey, the *X*_2_-*Y* relationship is weaker but still positive. In both countries, we revealed substantial causal interaction between *X*_3_ and *Y* (5%→0.35% in Turkey and 5%→0.31% in Vietnam). This result supports the estimations of Anderson and Nelgen [177], Giordani et al. [178], and Rude and An [179], who found that trade protectionism might trigger food inflation and thus aggravate food insecurity.

## 5. Conclusions

Irrespectively of any particular economic or food crisis, developing countries with their limited resources are more vulnerable to the deterioration of the macroeconomic environment. Food price volatility, no less food trade bans, is particularly detrimental to low-income countries where either a disruption of a supply chain or a contraction of economic access to staples may raise food conflicts. Before the COVID-19 outbreak, over two billion of the most impoverished people in the world spent up to 70% of their disposable income on food. The economic downturn stemmed from the pandemic may result in substantial growth of this figure, since in poorer countries, food demand is particularly linked to income [16]. In the past, both global (SARS and MERS) and local (Ebola, avian and swine flu) outbreaks had significant adverse effects on not only the health of people but also agricultural production and food consumption patterns across the developing world. The FAO expects hunger to increase in developing countries where the economy has slowed down or contracted due to the COVID-19 crisis [2]. There are threats to the access of the poor to food as a consequence of lost income from lockdowns, trade restrictions, food inflation, and currency depreciation. Most LDCs as well as many developing countries also suffer from underinvestment in public health, which may amplify the pandemic’s impacts [16].

This study is the very first try to assess the preliminary effects of the COVID-19 pandemic on the food security status of people across the developing world. In the cases of 45 LDCs and developing countries most vulnerable to food insecurity, the authors attempted to contribute to the nascent array of studies on trade and economic influences of the global health crisis over food availability and access to food and agricultural products. As distinguished from those few studies on COVID-19 effects on food supply chains that have been published so far, we revealed interactions between the number of COVID-19 cases and food security status of people across three groups of LDCs and developing economies. The consecutive application of the ARDL method, Yamamoto’s causality test, and variance decomposition allowed us to assess the impacts of foreign trade, inflation, and currency exchange on the number of people with insufficient food consumption during the global health crisis.

**Figure 2:**
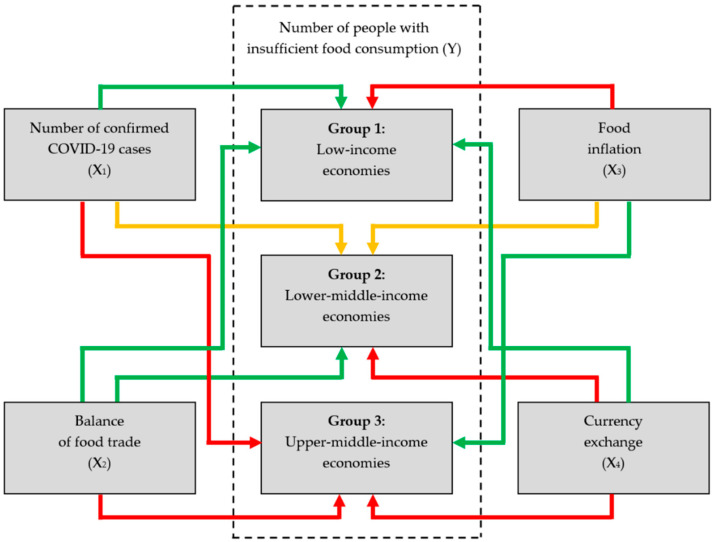
Summary of *X*_1–4_ effects on *Y* across three groups of countries. Note: Red = strong influence; yellow = medium influence; green = weak influence. Source: Authors’ development.

Three key findings have emerged from testing of the hypotheses in this study. First, the COVID-19 pandemic affects both the food security status of people and the stability of food supply chains in developing countries across the world. The effects are more perceptible in upper-middle-income economies than in LDCs given the deeper integration of the former in global supply chains and capital-intensive agricultural systems. Second, in lower-income developing countries, food security risks attributed to the emergence of the COVID-19 health crisis are mainly related to economic access to adequate food supply (represented by food inflation parameter). Third, in higher-income developing countries, availability-sided food security risks are more prevalent (represented by the parameters of food trade and currency exchange) (Figure 2).

Obviously, the estimations provided in this paper are rather rough. The study is built on a short array of data covering only six months that have passed from the start of the COVID-19 spread. Over time, seeding of new data on the number of new COVID-19 cases, dynamics of food trade balances, food inflation rates, and currency exchange volatilities will allow one to use the established methodology framework to obtain more well-grounded quantitative assessments of the pandemic’s impacts on food security. As more comprehensive data become available from the reports by WFP, FAO, WTO, and other organizations, the set of variables should be expanded to capture a multidimensional character of food security, including stability of food supply and utilization of food and agricultural products. We do not know whether the pandemic will decelerate by the fall of 2020 or whether the second wave will strike the world in 2021. It is yet hard to predict how effective the containment measures will be in slowing the spread of the virus. That is why the three-quarter variance decomposition projections presented in this study must be tested and adjusted continually to monitor the strengths of inter-variables causal interactions in the long-run. This will equip decision-makers with reliable estimations that may help to design coherent and effective policies to mitigate the impact of COVID-19 on food security across developing countries in various parts of the world.

## Figures and Tables

**Table 1:** Variables included in the study.

Index	Variable	Unit of Measure	Definition
*Y*	Number of people with insufficient food consumption	Millions of people	According to the World Food Programme (WFP) Food Consumption Score, people with a poor or borderline level of food consumption.
*X*_1_	Number of confirmed COVID-19 cases	Number of cases	Confirmed COVID-19 cases registered in a country per month.
*X*_2_	Balance of food trade	USD million	The value of exports of food and agricultural products less imports of food and agricultural production.
*X*_3_	Food inflation	Percentage	Month-on-month percentage change in the price of a standard basket of food as calculated from the national Consumer Price Index.
*X*_4_	Currency exchange	Monetary units	Price of a unit of domestic currency in terms of USD.

Source: Authors’ development.

**Table 2:** Food security and COVID-19: Group total numbers in January–June 2020.

Groups	Number of People with Insufficient Food Consumption						Number of Confirmed COVID-19 Cases					
	January 2020		June 2020		Change in January–June 2020		January 2020		June 2020		Change in January–June 2020	
	Mln People	% *	Mln People	% *	Mln People	p.p. **	cases	% *	Cases	% *	Cases	p.p. **
Group 1	180.552	36.735	186.030	37.849	+5.478	+1.115	1	-	84,334	0.017	+84,333	+0.017
Group 2	542.008	21.143	537.320	20.960	−4.688	−0.183	5	-	1,134,473	0.044	+1,134,468	+0.044
Group 3	57.940	13.759	51.530	12.237	−6.410	−1.522	1	-	986,952	0.234	986,951	+0.234

Note: * Portion in the total population of countries included in the group; ** change in percentage points; “+” parameter increment; “-” parameter decrement. Source: Authors’ development based on WFP’s Hunger Map portal [52].

**Table 3:** Study flow algorithm.

Number of Stage	Stage	Method	Results
1	Cointegration	Stationary test by Augmented Dickey-Fuller (ADF) and Phillips-Perron (PP) methods.	As certainty of cointegration between *Y* and *X*_1–4_.
2	Interaction	Autoregressive distributed lag (ARDL), fully modified ordinary least squares (FMOLS) and dynamic ordinary least squares (DOLS).	Identification of short and long-run interactions between the variables individually in forty-five countries, generalization of the results across three groups of economies by income and seven groups by region.
3	Causality	Toda–Yamamoto (TY) causality test.	Detection of the causality directions between the variables.
4	Relative strength	Variance decomposition method.	Exploration of the strengths of inter-variables causal interactions, verification of potential causality impacts till the first quarter of 2021.

Source: Authors’ development.

**Table 4:** Bound test results.

Group 1		Group 2		Group 3	
Country	F-Statistics	Country	F-Statistics	Country	F-Statistics
Afghanistan	12.85	Bangladesh	7.85	Algeria	11.67
Burkina Faso	5.17	Bolivia	11.99	Botswana	8.16
Chad	9.43	Cambodia	6.24	Colombia	6.04
Congo, Democratic Republic	7.11	Cameroon	8.12	Dominican Republic	22.85
Ethiopia	20.10	Cote d’Ivoire	13.55	Ecuador	18.03
Guinea	4.17	India	4.22	Guatemala	8.27
Haiti	23.65	Indonesia	9.03	Iran	5.70
Mali	18.49	Kenya	14.11	Iraq	12.63
Mozambique	6.16	Kyrgyzstan	5.48	Jordan	9.89
Nepal	3.21	Nigeria	6.93	Lebanon	14.55
Niger	17.55	Pakistan	4.19	Libya	8.28
Sierra Leone	10.38	Philippines	4.76	Namibia	10.07
Tajikistan	14.14	Tunisia	20.58	Peru	4.76
Tanzania	6.32	Vietnam	6.29	Sri Lanka	7.10
Yemen	9.47	Zambia	13.90	Turkey	9.12

Source: Authors’ development.

**Table 5:** Autoregressive distributed lag (ARDL) short-run estimates, group average.

Groups	Parameters	∆X_1_	∆X_2_	∆X_3_	∆X_4_	ECM
Group 1	Coefficient	0.0432	0.1004	0.3678	−0.0402	−0.1137
	t-stat	1.8935	2.8377	1.1605	2.7808	−1.1794
	Prob	0.1063	0.1083	0.1619	0.1626	0.0034
Group 2	Coefficient	0.2241	0.1872	0.1329	0.2232	0.0892
	t-stat	1.5251	1.5513	1.5460	0.8845	0.5580
	Prob	0.0611	0.1653	0.1537	0.1507	0.0234
Group 3	Coefficient	0.0441	0.2731	−0.0372	0.1458	−0.0380
	t-stat	2.4477	2.4722	0.4654	0.3367	0.1839
	Prob	0.0435	0.0825	0.0547	0.1120	0.0181

*X*_1_ = number of confirmed COVID-19 cases; *X*_2_ = balance of food trade; *X*_3_ = food inflation; *X*_4_ = currency exchange. Source: Authors’ development.

**Table 6:** Fully modified ordinary least squares (FMOLS) and Dynamic ordinary least squares (DOLS) tests results and Autoregressive distributed lag (ARDL) long-run estimates, group average.

Groups	Parameters	X_1_	X_2_	X_3_	X_4_	Constant
Group 1	ARDL coefficient	0.0412	0.0933	0.3638	−0.0214	−2.7583
	ARDL t-stat	2.1187	2.8695	1.3274	2.9730	−1.1813
	FMOLS coefficient	0.0358	0.0390	0.3836	0.0112	−2.3624
	FMOLS t-stat	1.8692	2.0291	1.3168	2.5642	−1.2765
	DOLS coefficient	0.0364	0.0497	0.3895	−0.0207	−2.6475
	DOLS t-stat	1.9093	2.4646	1.3746	2.6088	−1.0205
Group 2	ARDL coefficient	0.2192	0.2348	0.1627	0.2510	0.5579
	ARDL t-stat	1.9379	1.7600	1.6597	0.9202	0.5980
	FMOLS coefficient	0.2211	0.2542	0.1783	0.2551	0.4373
	FMOLS t-stat	1.9656	1.6926	1.2906	1.7336	0.8624
	DOLS coefficient	0.2209	0.2348	0.1630	0.2507	0.3867
	DOLS t-stat	1.8771	1.7220	1.5830	0.9665	0.6168
Group 3	ARDL coefficient	0.0411	0.3182	−0.0331	0.2105	1.0449
	ARDL t-stat	2.9831	3.0380	0.6251	0.5951	0.4289
	FMOLS coefficient	0.0505	0.3349	−0.0066	0.2321	1.1124
	FMOLS t-stat	2.8097	3.0461	0.6724	0.5461	0.4539
	DOLS coefficient	0.0460	0.3196	−0.0213	0.2173	1.0031
	DOLS t-stat	2.9605	3.0479	0.6242	0.6168	0.4228

*X*_1_ = number of confirmed COVID-19 cases; *X*_2_ = balance of food trade; *X*_3_ = food inflation; *X*_4_ = currency exchange. Source: Authors’ development.

**Table 7:** Toda-Yamamoto (TY) causality test results, group average.

Groups	Parameters	$Y\to X_{1}$	$X_{1}\to Y$	$Y\to X_{2}$	$X_{2}\to Y$	$Y\to X_{3}$	$X_{3}\to Y$	$Y\to X_{4}$	$X_{4}\to Y$
Group 1	Test statistics	3.17	7.84	2.15	7.31	2.09	10.11	1.80	5.67
	P value	0.40	0.19	0.52	0.14	0.53	0.16	0.61	0.31
Group 2	Test statistics	0.52	6.03	0.58	5.87	0.80	6.95	0.51	3.68
	P value	0.60	0.12	0.57	0.11	0.52	0.06	0.65	0.24
Group 3	Test statistics	0.33	9.29	0.90	9.03	1.09	5.12	0.71	5.06
	P value	0.73	0.09	0.45	0.05	0.37	0.13	0.51	0.18

*Y* = number of people with insufficient food consumption; *X*_1_ = number of confirmed COVID-19 cases; *X*_2_ = balance of food trade; *X*_3_ = food inflation; *X*_4_ = currency exchange. Source: Authors’ development.

**Table 8:** Variance decomposition of *Y* over a nine-period (three quarters) horizon, group average.

Groups	Periods	Standard Error	Y	X_1_	X_2_	X_3_	X_4_
Group 1	September 2020	0.009763	94.603805	1.042113	1.627254	1.742278	0.984550
	December 2020	0.014191	88.846093	2.563652	2.744643	3.416632	2.428979
	March 2021	0.019954	82.571860	3.858422	4.232789	5.307862	4.029067
Group 2	September 2020	0.008970	94.760896	1.092635	1.181600	2.105061	0.859808
	December 2020	0.013314	88.052805	2.499151	2.535821	4.752614	2.159609
	March 2021	0.018051	82.158020	3.442063	3.901844	7.457695	3.040378
Group 3	September 2020	0.012420	94.571379	1.733111	2.041619	0.805161	0.848729
	December 2020	0.018622	87.448534	3.409666	4.909030	1.569857	2.662913
	March 2021	0.024269	81.049395	5.149831	7.403395	2.325381	4.071998

*Y* = number of people with insufficient food consumption; *X*_1_ = number of confirmed COVID-19 cases; *X*_2_ = balance of food trade; *X*_3_ = food inflation; *X*_4_ = currency exchange. Source: Authors’ development.

## Supplementary Materials

The following are available online at https://www.mdpi.com/1660-4601/17/16/5775/s1. Table S1: ADF and PP tests results, Group 1, Table S2: ADF and PP tests results, Group 2, Table S3: ADF and PP tests results, Group 3, Table S4: ARDL short-run estimates, Group 1, Table S5: ARDL short-run estimates, Group 2, Table S6: ARDL short-run estimates, Group 3, Table S7: FMOLS and DOLS tests results and ARDL long-run estimates, Group 1, Table S8: FMOLS and DOLS tests results and ARDL long-run estimates, Group 2, Table S9: FMOLS and DOLS tests results and ARDL long-run estimates, Group 3, Table S10: TY causality test results, Group 1, Table S11: TY causality test results, Group 2, Table S12: TY causality test results, Group 3, Table S13: Variance decomposition of *Y* over a nine periods (three quarters) horizon, Group 1, Table S14: Variance decomposition of *Y* over a nine periods (three quarters) horizon, Group 2, Table S15: Variance decomposition of *Y* over a nine periods (three quarters) horizon, Group 3.

## References

[1] United Nations Sustainable Development Goals. https://www.un.org/sustainabledevelopment/sustainable-development-goals/.

[2] Food and Agriculture Organization of the United Nations, International Fund for Agricultural Development, United Nations Children’s Fund, World Food Programme, World Health Organization (2019). The State of Food Security and Nutrition in the World 2019. Safeguarding against Economic Slowdowns and Downturns.

[3] Food and Agriculture Organization of the United Nations, International Fund for Agricultural Development, United Nations Children’s Fund, World Food Programme, World Health Organization (2020). The State of Food Security and Nutrition in the World 2020. Transforming Food Systems for Affordable Healthy Diets.

[4] Food and Agriculture Organization of the United Nations (1992). Food, Nutrition, and Agriculture.

[5] Vos R., Martin W., Laborde D. How Much Will Global Poverty Increase Because of COVID-19?. https://www.ifpri.org/blog/how-much-will-global-poverty-increase-because-covid-19.

[6] John Hopkins University Coronavirus Resource Center. https://coronavirus.jhu.edu/map.html.

[7] Benton T. (2020). COVID-19 and Disruptions to Food Systems. Agric. Hum. Values.

[8] Toffolutti V., Stuckler D., McKee M. (2020). Is the COVID-19 Pandemic Turning into a European Food Crisis?. Eur. J. Public Health.

[9] Blay-Palmer A., Carey R., Valette E., Sanderson M. (2020). Post COVID 19 and Food Pathways to Sustainable Transformation. Agric. Hum. Values.

[10] Welsh C. Covid-19 and Food Security. https://www.csis.org/programs/global-food-security-program/covid-19-and-food-security.

[11] Devereux S., Béné C., Hoddinott J. (2020). Conceptualising COVID-19′s Impacts on Household Food Security. Food Secur..

[12] Bakalis S., Valdramidis V., Argyropoulos D., Ahrne L., Chen J., Cullen P., Cummins E., Datta A., Emmanouilidis C., Foster T. (2020). Perspectives from CO+RE: How COVID-19 Changed Our Food Systems and Food Security Paradigms. Curr. Res. Food Sci..

[13] Farrell P., Thow A.M., Wate J.T., Nonga N., Vatucawaqa P., Brewer T., Sharp M., Farmery A., Trevena H., Reeve E. (2020). COVID-19 and Pacific Food System Resilience: Opportunities to Build a Robust Response. Food Secur..

[14] World Food Programme COVID-19 Will Double Number of People Facing Food Crises Unless Swift Action Is Taken. https://www.wfpusa.org/news-release/covid-19-will-double-number-of-people-facing-food-crises-unless-swift-action-is-taken/.

[15] World Trade Organization The COVID-19 Pandemic and Trade-Related Developments in LDCs. https://www.wto.org/english/tratop_e/covid19_e/ldcs_report_e.pdf.

[16] Food and Agriculture Organization of the United Nations Novel Coronavirus (COVID-19). http://www.fao.org/2019-ncov/q-and-a/impact-on-food-and-agriculture/en/.

[17] Laborde D., Mamun A., Parent M. (2020). COVID-19 Food Trade Policy Tracker [Dataset].

[18] Headey D. (2011). Rethinking the Global Food Crisis: The Role of Trade Shocks. Food Policy.

[19] Hendrix C.S. Wrong Tools, Wrong Time: Food Export Bans in the Time of COVID-19. https://www.piie.com/blogs/realtime-economic-issues-watch/wrong-tools-wrong-time-food-export-bans-time-covid-19.

[20] Martin W., Anderson K. (2011). Export Restrictions and Price Insulation during Commodity Price Booms.

[21] Puma M., Bose S., Chon S.Y., Cook B. (2015). Assessing the Evolving Fragility of the Global Food System. Environ. Res. Lett..

[22] Lima T., Dias A., Palma I., Amorim L. COVID-19 and Food Insecurity: What Is the Role of International Cooperation?. https://www.agrariansouth.org/2020/04/18/covid-19-and-food-insecurity-what-is-the-role-of-international-cooperation/.

[23] Food and Agriculture Organization of the United Nations Daily Food Prices Monitor. https://datalab.review.fao.org/dailyprices.html.

[24] World Food Programme The Market Monitor. https://www.wfp.org/publications/market-monitor.

[25] World Trade Organization Members Discuss Impact of COVID-19 on Developing Economies’ Participation in World Trade. https://www.wto.org/english/news_e/news20_e/devel_26may20_e.htm.

[26] Ritson C. (1980). Self-Sufficiency and Food Security.

[27] Luan Y., Cui X., Ferrat M. (2013). Historical Trends of Food Self-Sufficiency in Africa. Food Secur..

[28] Lee R. (2007). Food Security and Food Sovereignty.

[29] Smutka L., Spicka J., Ishchukova N., Selby R. (2016). Agrarian Import Ban and Its Impact on Russian and European Union Agrarian Trade Performance. Agric. Econ. Czech..

[30] Erokhin V. (2017). Self-Sufficiency versus Security: How Trade Protectionism Challenges the Sustainability of the Food Supply in Russia. Sustainability.

[31] Food and Agriculture Organisation of the United Nations Implications of Economic Policy for Food Security: A Training Manual. http://www.fao.org/docrep/004/x3936e/x3936e03.htm.

[32] Shattuck A., Schiavoni C.M., VanGelder Z. (2015). Translating the Politics of Food Sovereignty: Digging into Contradictions, Uncovering New Dimensions. Globalizations.

[33] Kofman J. (1997). Economic Nationalism and Development: Central and Eastern Europe between the Two World Wars.

[34] Wegren S.K., Elvestad C. (2018). Russia’s Food Self-Sufficiency and Food Security: An Assessment. Post-Communist Econ..

[35] Meskhia I. (2016). Food Security Problems in Post Soviet Georgia. Ann. Agrar. Sci..

[36] Clapp J. (2017). Food Self-Sufficiency: Making Sense of It, and When It Makes Sense. Food Policy.

[37] Saidi A., Diouri M. (2017). Food Self-Sufficiency under the Green-Morocco Plan. J. Exp. Biol. Agric. Sci..

[38] Squires V.R., Gaur M.K., Squires V., Gaur M. (2020). Agricultural Productivity and Food Security. Food Security and Land Use Change under Conditions of Climatic Variability.

[39] Faraco R., Silva C., Amorim W., Silveira A., Neiva S., Baltazar J. (2016). Food Security, Agriculture and Climate Change Mitigation Strategies: A Scientific Production Panorama. Sch. Int. J. Multidiscip. Allied Stud..

[40] Timmer C.P. (2000). The Macro Dimensions of Food Security: Economic Growth, Equitable Distribution, and Food Price Stability. Food Policy.

[41] Manap N.M.A., Ismail N.W. (2019). Food Security and Economic Growth. Int. J. Mod. Trends Soc. Sci..

[42] Lake I.R., Hooper L., Abdelhamid A., Bentham G., Boxall A.B.A., Draper A., Fairweather-Tait S., Hulme M., Hunter P.R., Nichols G. (2012). Climate Change and Food Security: Health Impacts in Developed Countries. Environ. Health Perspect..

[43] Hastrup K., Rieffestahl A.M., Olsen A., Singer M. (2016). Food Security: Health and Environmental Concerns in the North. A Companion to the Anthropology of Environmental Health.

[44] Kinsey B.H., Mazibuko Z. (2019). Syndemics, Food Security, Health, and Local Environments: Chronic Undernutrition in Zimbabwe. Epidemics and the Health of African Nations.

[45] Committee for the Coordination of Statistical Activities (2020). How COVID-19 Is Changing the World: A Statistical Perspective.

[46] Unhale S.S., Ansar Q.B., Gajghane V., Bharudkar S.S., Gadekar P.P., Biyani K.R. (2020). Impact of COVID-19 on Food Safety and Food Security. World J. Adv. Healthc. Res..

[47] Mukiibi E. (2020). COVID-19 and the State of Food Security in Africa. Agric. Hum. Values.

[48] Hussein H., Greco F. (2020). How Will the COVID-19 Pandemic Impact Food Security and Virtual Water “Trade”?. Future Food J. Food Agric. Soc..

[49] Rahman S., Hossain I., Mullick A.R., Khan M.H. (2020). Food Security and the Coronavirus Disease 2019 (COVID-19): A Systemic Review. J. Med. Sci. Clin. Res..

[50] Zurayk R. (2020). Pandemic and Food Security: A View from the Global South. J. Agric. Food Syst. Community Dev..

[51] Dev D., Kabir K. (2020). COVID-19 and Food Security in Bangladesh: A Chance to Look Back at What Is Done and What Can Be Done. J. Agric. Food Syst. Community Dev..

[52] World Food Programme Hunger Map. https://hungermap.wfp.org/.

[53] Huseynov R. (2019). Multidimensional Determinants of National Food Security in Azerbaijan: An Application of the ARDL Approach. Probl. World Agric..

[54] Trading Economics Countries. https://tradingeconomics.com/countries.

[55] United Nations Conference on Trade and Development Data Center. https://unctadstat.unctad.org/wds/ReportFolders/reportFolders.aspx?sCS_ChosenLang=en.

[56] World Bank World Bank Country and Lending Groups. https://datahelpdesk.worldbank.org/knowledgebase/articles/906519-world-bank-country-and-lending-groups.

[57] World Bank The World Bank Atlas Method–Detailed Methodology. https://datahelpdesk.worldbank.org/knowledgebase/articles/378832-what-is-the-world-bank-atlas-method.

[58] Vos R., Martin W., Laborde D. As COVID-19 Spreads, No Major Concern for Global Food Security yet. https://www.ifpri.org/blog/covid-19-spreads-no-major-concern-global-food-security-yet.

[59] Smith L.C., El Obeid A.E., Jensen H.H. (2000). The Geography and Causes of Food Insecurity in Developing Countries. Agric. Econ..

[60] Tomkins A.J. (2012). Combating Food Shortages in Least Developed Countries: Current Development Assistance Approaches. Law Dev. Rev..

[61] Koirala N.P., Koirala D.P. (2014). Political Economy of Food Security in Least Developed Nations: A Review. J. Agric. Environ..

[62] Ritchie H., Reay D., Higgins P. (2018). Sustainable Food Security in India–Domestic Production and Macronutrient Availability. PLoS ONE.

[63] Samaratunga P.A., Mittal S., Sethi D. (2011). Multiple Facets of Food (in) Security in Sri Lanka: An Input to Food Policy. Policy Options to Achieve Food Security in South Asia.

[64] Erokhin V. (2018). Establishing Food Security and Alternatives to International Trade in Emerging Economies.

[65] Thome K., Meade B., Rosen S., Beghin J.C., Schmitz A., Kennedy P.L., Schmitz T.G. (2017). Assessing Food Security in Ethiopia. World Agricultural Resources and Food Security (Frontiers of Economics and Globalization, Vol. 17).

[66] Power E.M. (2005). Determinants of Healthy Eating Among Low-Income Canadians. Can. J. Public Health.

[67] Sonnino R., Faus A.M., Maggio A. (2014). Sustainable Food Security: An Emerging Research and Policy Agenda. Int. J. Sociol. Agric. Food.

[68] Erokhin V., Gao T. (2020). Handbook of Research on Globalized Agricultural Trade and New Challenges for Food Security.

[69] Smith V.H., Glauber J.W. (2020). Trade, Policy, and Food Security. Agric. Econ..

[70] Yeung M., Kerr W., Coomber B., Lantz M., McConnell A., Yeung M., Kerr W., Coomber B., Lantz M., McConnell A. (2017). The Importance of Trade for Food Security. Declining International Cooperation on Pesticide Regulation.

[71] Chang T., Lee K.-C., Kang S.-C., Liu W.-C. (2008). Is Per Capita Real GDP Stationary in Latin American Countries? Evidence from a Panel Stationary Test with Structural Breaks. Econ. Bull..

[72] Bahmani-Oskooee M., Chang T., Wu T. (2014). Revisiting Purchasing Power Parity in African Countries: Panel Stationary Test with Sharp and Smooth Breaks. Appl. Financ. Econ..

[73] Kosicka E., Kozłowski E., Mazurkiewicz D. (2015). The Use of Stationary Tests for Analysis of Monitored Residual Processes. Eksploat. I Niezawodn. Maint. Reliab..

[74] Dickey D.A., Fuller W.A. (1981). Likelihood Ratio Statistics for Autoregressive Time Series with a Unit Root. Econometrica.

[75] Phillips P.C., Perron P. (1988). Testing for a Unit Root in Time Series Regression. Biometrika.

[76] Herwartz H., Reimers H.E. (2006). Panel Nonstationary Tests of the Fisher Hypothesis: An Analysis of 114 Economies during the Period 1960–2004. Appl. Econom. Int. Dev..

[77] Chang T., Chiang G., Zhang Y. (2009). Is Volume Index of GDP Per Capita Stationary in OECD Countries? Panel Stationary Tests with Structural Breaks. Econ. Bull..

[78] Ranjbar O., Lee C.-C., Chang T., Chen M.-P. (2013). Income Convergence in African Countries: Evidence from a Stationary Test with Multiple Structural Breaks. South Afr. J. Econ..

[79] Su C.-W., Chang H.-L., Zhu M.-N. (2012). Flexible Fourier Stationary Test in Purchasing Power Parity for African Countries. Appl. Econ..

[80] Hoarau J.-F. (2008). Testing PPP for Central American Real Exchange Rates. Evidence from New Panel Data Stationary Tests with Structural Breaks. Econ. Bull..

[81] Aliyev K., Dehning B., Nadirov O. (2016). Modelling the Impact of Fiscal Policy on Non-Oil GDP in a Resource Rich Country: Evidence from Azerbaijan. Acta Univ. Agric. Et Silvic. Mendel. Brun..

[82] Humbatova S.I., Tanriverdiev S.M., Mammadov I.N., Hajiyev N.G.O. (2020). Impact of Investment on GDP and Non-Oil GDP in Azerbaijan. Entrep. Sustain. Issues.

[83] Naseem S., Tong G., Kashif U. (2020). Exploring the Impact of Energy Consumption, Food Security on CO2 Emissions: A Piece of New Evidence from Pakistan. Int. Energy J..

[84] Kuo B.-S., Mikkola A. (1999). Re-Examining Long-Run Purchasing Power Parity. J. Int. Money Financ..

[85] Gunduz M. (2020). The Link between Unemployment and Industrial Production: The Fourier Approach with Structural Breaks. Econ. Soc. Chang. Facts Trends.

[86] Tsen W. (2019). Real Exchange Rate Returns and Real Stock Price Returns in the Stock Market of Malaysia. Singap. Econ. Rev..

[87] Culver S.E., Papell D.H. (1999). Long-Run Purchasing Power Charity with Short-Run Data: Evidence with a Null Hypothesis of Stationarity. J. Int. Money Financ..

[88] Chen Y., Pun C. (2019). A Bootstrap-Based KPSS Test for Functional Time Series. J. Multivar. Anal..

[89] Jin S., Phillips P. (2002). The KPSS Test with Seasonal Dummies. Econ. Lett..

[90] Hadri K., Rao Y. (2009). KPSS Test and Model Misspecifications. Appl. Econ. Lett..

[91] Pesaran M.H., Shin Y., Smith R.J. (2001). Bounds Testing Approaches to the Analysis of Level Relationships. J. Appl. Econom..

[92] Öztürk S., Özdil S. (2020). Investigation of the Relationship between Migration, Unemployment and Growth in the OECD Countries with Panel ARDL Technique. Yönetim Ve Ekon..

[93] Elian M., Sawalha N., Bani-Mustafa A. (2020). Revisiting the FDI-Growth Nexus: ARDL Bound Test for BRICS Standalone Economies. Mod. Appl. Sci..

[94] Appiah M., Li F., Korankye B. (2019). Foreign Investment and Growth in Emerging Economies: Panel ARDL Analysis. J. Econ. Bus. Account. Ventur..

[95] Raghuramapatruni R., Chaitanya R.V.S. (2020). An Appraisal of the Impact of International Trade on Economic Growth of India–through the ARDL Approach. Int. J. Econ. Bus. Adm..

[96] Oluwafemi I.J., Laseinde O.T. (2019). Macroeconomic as Basis of Economic Growth: An ARDL Approach. J. Phys. Conf. Ser..

[97] Adebayo T.S. (2020). New Insights into Export-Growth Nexus: Wavelet and Causality Approaches. Asian J. Econ. Bus. Account..

[98] Alqaralleh H. (2020). Stock Return-Inflation Nexus; Revisited Evidence Based on Nonlinear ARDL. J. Appl. Econ..

[99] Karahan Ö., Çolak O., Janowicz-Lomott M., Łyskawa K., Polychronidou P., Karasavvoglou A. (2020). Inflation and Economic Growth in Turkey: Evidence from a Nonlinear ARDL Approach. Economic and Financial Challenges for Balkan and Eastern European Countries.

[100] Musa A.B., Danlami I.A., Elijah S. (2019). The Asymmetric Effect of Currency Devaluation on Inflation in Malaysia; Evidence from Non-Linear ARDL. Int. J. Recent Technol. Eng..

[101] Ebrahimi P., Alipour H., Gholampour A., Ahmadi M. (2019). Social Networks, Exchange Rate Fluctuation, and Economic Growth: ARDL Approach. Tékhne–Rev. Appl. Manag. Stud..

[102] Omotayo O.H., Ayomitunde A.T., Afolakemi A.O., Aromoke O.O. (2019). Health, Agricultural Expenditure and Economic Growth in Nigeria: ARDL and ECM Approach. Int. J. New Econ. Soc. Sci..

[103] Phillips P.C., Hansen B.E. (1990). Statistical Inference in Instrumental Variables Regression with I (1) Processes. Rev. Econ. Stud..

[104] Yuzbashkandi S.S., Sadi M.A. (2020). Petroleum Production Impacts on the Economic Growth of the OPEC Countries: Panel ARDL Approach. Sn Appl. Sci..

[105] Narayan S., Narayan P.K. (2004). Determinants of Demand for Fiji’s Exports: An Empirical Investigation. Dev. Econ..

[106] Abu N. (2019). Inflation and Unemployment Trade-off: A Re-Examination of the Phillips Curve and Its Stability in Nigeria. Contemp. Econ..

[107] Priyankara E.A.C. (2018). The Long-run Effect of Services Exports on Total Factor Productivity Growth in Sri Lanka: Based on ARDL, FMOLS, CCR, and DOLS Approaches. Int. J. Acad. Res. Bus. Soc. Sci..

[108] Bashier A.A., Siam A.J. (2014). Immigration and Economic Growth in Jordan: FMOLS Approach. Int. J. Humanit. Soc. Sci. Educ..

[109] Stock J.H., Watson M. (1993). A Simple Estimator of Cointegrating Vectors in Higher Order Integrated Systems. Econometrica.

[110] Kurozumi E., Hayakawa K. (2009). Asymptotic Properties of the Efficient Estimators for Cointegrating Regression Models with Serially Dependent Errors. J. Econom..

[111] Herzer D., Nowak-Lehmann F., Siliverstovs B. (2006). Export-Led Growth in Chile: Assessing the Role of Export Composition in Productivity Growth. Dev. Econ..

[112] Pasha A., Ramzan M. (2019). Asymmetric Impact of Economic Value-Added Dynamics on Market Value of Stocks in Pakistan Stock Exchange, a New Evidence from Panel Cointegration, FMOLS and DOLS. Cogent Bus. Manag..

[113] Guan J., Kirikkaleli D., Bibi A., Zhang W. (2020). Natural Resources Rents Nexus with Financial Development in the Presence of Globalization: Is the “Resource Curse” Exist or Myth?. Resour. Policy.

[114] Yue S., Munir I.U., Hyder S., Nassani A.A., Abro M.M.Q., Zaman K. (2020). Sustainable Food Production, Forest Biodiversity and Mineral Pricing: Interconnected Global Issues. Resour. Policy.

[115] Rahman Z.U., Khattak S.I., Ahmad M., Khan A. (2020). A Disaggregated-Level Analysis of the Relationship among Energy Production, Energy Consumption and Economic Growth: Evidence from China. Energy.

[116] Toda H.Y., Yamamoto T. (1995). Statistical Inference in Vector Autoregressions with Possibly Integrated Processes. J. Econom..

[117] Pantamee A.A., Yola A.T., Masud A. (2020). The Nexus between Tax Revenue and Government Expenditure in Nigeria; Evidence from Toda–Yamamoto Causality Test. Int. J. Innov. Creat. Chang..

[118] Bilgehan T. (2018). Toda–Yamamoto Causality between E7 Countries Stock Markets. Econ. Mark. Commun. Rev..

[119] Sankaran A., Kumar S., Arjun K., Das M. (2019). Estimating the Causal Relationship between Electricity Consumption and Industrial Output: ARDL Bounds and Toda–Yamamoto Approaches for Ten Late Industrialized Countries. Heliyon.

[120] Rana R., Sharma M. (2020). Dynamic Causality among FDI, Economic Growth and CO2 Emissions in India with Open Markets and Technology Gap. Int. J. Asian Bus. Inf. Manag..

[121] Rana R., Sharma M. (2019). Dynamic Causality Testing for EKC Hypothesis, Pollution Haven Hypothesis and International Trade in India. J. Int. Trade Econ. Dev..

[122] Wang J., Ngene G. (2018). Symmetric and Asymmetric Nonlinear Causalities between Oil Prices and the U.S. Economic Sectors. Rev. Quant. Financ. Account..

[123] Hayashi K., Bentler P., Yuan K., Rao C.R., Miller J.P., Rao D.C. (2010). Structural Equation Modeling. Essential Statistical Methods for Medical Statistics.

[124] Lemonte A. (2016). The Gradient Test: Another Likelihood-Based Test.

[125] Zhang Y., Cheng Z., He Q. (2020). Time Lag Analysis of FDI Spillover Effect: Evidence from the Belt and Road Developing Countries Introducing China’s Direct Investment. Int. J. Emerg. Mark..

[126] Mao X., Yang A., Peng C., Shang P. (2020). Analysis of Economic Growth Fluctuations Based on EEMD and Causal Decomposition. Phys. A Stat. Mech. Its Appl..

[127] Chan J., Eisenstat E., Strachan R. (2020). Reducing the State Space Dimension in Large TVP-VAR. J. Econom..

[128] Mouloudj K., Bouarar A.C., Fechit H. (2020). The Impact of COVID-19 Pandemic on Food Security. Les. Cah. Du Cread.

[129] Poudel P.B., Poudel M.R., Gautam A., Phuyal S., Tiwari C.K., Bashyal N., Bashyal S. (2020). COVID-19 and Its Global Impact on Food and Agriculture. J. Biol. Today’s World.

[130] Siche R. (2020). What Is the Impact of COVID-19 Disease on Agriculture?. Sci. Agropecu..

[131] Ceylan R.F., Ozkan B. (2020). The Economic Effects of Epidemics: From SARS and MERS to COVID-19. Res. J. Adv. Humanit..

[132] Kodish S.R., Bio F., Oemcke R., Conteh J., Beauliere J.M., Pyne-Bailey S., Rohner F., Ngnie-Teta I., Jalloh M.B., Wirth J.P. (2019). A Qualitative Study to Understand How Ebola Virus Disease Affected Nutrition in Sierra Leone: A Food Value-Chain Framework for Improving Future Response Strategies. PLoS Negl. Trop. Dis..

[133] Wernery U., Woo P.C.Y. (2019). Middle East Respiratory Syndrome: Making the Case for Surveillance of Transboundary Coronaviruses in the Middle East. Rev. Sci. Et Tech. Off. Int. Des Epizoot..

[134] Duonamou L., Konate A., Djossou S.D., Mensah G.A., Xu J., Humle T. (2020). Consumer Perceptions and Reported Wild and Domestic Meat and Fish Consumption Behavior During the Ebola Epidemic in Guinea, West Africa. PeerJ.

[135] Petersen E., Koopmans M., Go U., Hamer D., Petrosillo N., Castelli F., Storgaard M., Al Khalili S., Simonsen L. (2020). Comparing SARS-CoV-2 with SARS-CoV and Influenza Pandemics. Lancet Infect. Dis..

[136] Khatib M.N., Gaidhane S., Khatib M., Ahmed M., Gaidhane A., Syed Z.Q. (2020). SARS-CoV and SARS-CoV-2: Similar Viruses with Different Trajectories. Wutan Huatan Jisuan Jishu.

[137] Alfaraj S.H., Al-Tawfiq J.A., Assiri A.Y., Alzahrani N.A., Alanazi A.A., Memish Z.A. (2019). Clinical Predictors of Mortality of Middle East Respiratory Syndrome Coronavirus (MERS-CoV) Infection: A Cohort Study. Travel Med. Infect. Dis..

[138] Bhunnoo R. (2019). The Need for a Food-Systems Approach to Policy Making. Lancet.

[139] Patz J.A., Campbell-Lendrum D., Holloway T., Foley J.A. (2005). Impact of Regional Climate Change on Human Health. Nature.

[140] Alirol E., Getaz L., Stoll B., Chappuis F., Loutan L. (2011). Urbanisation and Infectious Diseases in a Globalised World. Lancet Infect. Dis..

[141] Reardon T., Bellemare M., Zilberman D. How COVID-19 May Disrupt Food Supply Chains in Developing Countries. https://www.ifpri.org/blog/how-covid-19-may-disrupt-food-supply-chains-developing-countries.

[142] Woertz E. (2020). Wither the Self-Sufficiency Illusion? Food Security in Arab Gulf States and the Impact of COVID-19. Food Secur..

[143] Hasöksüz M., Kiliç S., Saraç F. (2020). Coronaviruses and SARS-COV-2. Turk. J. Med. Sci..

[144] Silva-Jaimes M.I. (2020). SARS-CoV-2 and Other Emerging Viruses and Their Relationship to Safety in the Food Chain. Sci. Agropecu..

[145] Shahidi F. (2020). Does COVID-19 Affect Food Safety and Security? A Summary Report on the Extraordinary Scientific Roundtable of IUFoST-CIFST on March 21, 2020. J. Food Bioact..

[146] (2020). Food and Agriculture Organization of the United Nations.

[147] Shilomboleni H. (2020). COVID-19 and Food Security in Africa: Building More Resilient Food Systems [version 1; peer review: 2 approved]. AAS Open Res..

[148] Janssens C., Havlík P., Krisztin T., Baker J., Frank S., Hasegawa T., Leclère D., Ohrel S., Ragnauth S., Schmid E. (2020). Global Hunger and Climate Change Adaptation Through International Trade. Nat. Clim. Chang..

[149] Freund C., Özden C. (2008). Trade Policy and Loss Aversion. Am. Econ. Rev..

[150] Food and Agriculture Organization of the United Nations FAO Food Price Index. http://www.fao.org/worldfoodsituation/foodpricesindex/en/.

[151] Berkowitz S.A., Basu S., Meigs J.B., Seligman H.K. (2018). Food Insecurity and Health Care Expenditures in the United States, 2011–2013. Health Serv. Res..

[152] Gundersen C., Ziliak J.P. (2015). Food Insecurity and Health Outcomes. Health Aff..

[153] Garcia S.P., Haddix A., Barnett K. (2018). Incremental Health Care Costs Associated with Food Insecurity and Chronic Conditions among Older Adults. Prev. Chronic Dis..

[154] Niles M.T., Bertmann F., Belarmino E.H., Wentworth T., Biehl E., Neff R. (2020). The Early Food Insecurity Impacts of COVID-19. Nutrients.

[155] Esturk O., Ören M.N. (2013). Impact of Household Socio-Economic Factors on Food Security: Case of Adana. Pak. J. Nutr..

[156] Fawzi I., Qurani Z., Rahmasary N. (2020). COVID-19: Implication to Food Security.

[157] Yuen K.F., Wang X., Ma F., Li K.X. (2020). The Psychological Causes of Panic Buying Following a Health Crisis. Int. J. Environ. Res. Public Health.

[158] Koptyug E. Sales Revenue Change for Select Products in Food Retail due to Panic Buying during the Coronavirus (COVID-19) Pandemic in Germany in 2020. https://www.statista.com/statistics/1104200/coronavirus-panic-buying-food-retail-sales-revenue-change-germany/.

[159] Bench A. COVID-19: As Panic-Buying Increases, Expert Says Supply Chain Should Hold Up. https://globalnews.ca/news/6651464/covid-19-panic-buying-shortages/.

[160] Reeves A., Loopstra R., Stuckler D. (2017). The Growing Disconnect between Food Prices and Wages in Europe: Cross-National Analysis of Food Deprivation and Welfare Regimes in Twenty-One EU Countries, 2004–2012. Public Health Nutr..

[161] Erokhin V. (2017). Factors Influencing Food Markets in Developing Countries: An Approach to Assess Sustainability of the Food Supply in Russia. Sustainability.

[162] Otsuka K. (2013). Food Insecurity, Income Inequality, and the Changing Comparative Advantage in World Agriculture. Agric. Econ..

[163] Elbushra A.A., Ahmed A.E. (2020). Food Security in Sudan: A Historical Analysis of Food Availability. Iraqi J. Agric. Sci..

[164] Kinsey E., Kinsey D., Rundle A. (2020). COVID-19 and Food Insecurity: An Uneven Patchwork of Responses. J. Urban. Health.

[165] Kinsey E., Oberle M., Dupuis R., Cannuscio C.C., Hillier A. (2019). Food and Financial Coping Strategies during the Monthly Supplemental Nutrition Assistance Program Cycle. SSM–Popul. Health.

[166] Deuss A. (2017). Impact of Agricultural Export Restrictions on Prices in Importing Countries.

[167] Wood S.A., Smith M.R., Fanzo J., Remans R., DeFries R.S. (2018). Trade and the Equitability of Global Food Nutrient Distribution. Nat. Sustain..

[168] Frankenberg E., Thomas D., Barrett C.B., Carter M.R., Chavas J.-P. (2019). Human Capital and Shocks: Evidence on Education, Health, and Nutrition. The Economics of Poverty Traps.

[169] Djuric I., Götz L., Glauben T. (2015). Are Export Restrictions an Effective Instrument to Insulate Domestic Prices against Skyrocketing World Market Prices? The Wheat Export Ban in Serbia. Agribus. Int. J..

[170] Johnson D.G. (1975). World Agriculture, Commodity Policy, and Price Variability. Am. J. Agric. Econ..

[171] Ivanic M., Martin W. (2008). Implications of Higher Global Food Prices for Poverty in Low-Income Countries. Agric. Econ..

[172] Bouët A., Laborde D. (2010). Economics of Export Taxation in a Context of Food Crisis: A Theoretical and CGE Approach Contribution.

[173] Timmer C.P. (2010). Reflections on Food Crises Past. Food Policy.

[174] Robles M., Torero M., von Braun J. (2009). When Speculation Matters.

[175] Dawe D., Timmer C.P. (2012). Why Stable Food Prices Are a Good Thing: Lessons from Stabilizing Rice Prices in Asia. Glob. Food Secur..

[176] Abbott P. (2011). Export Restrictions as Stabilization Responses to Food Crisis. Am. J. Agric. Econ..

[177] Anderson K., Nelgen S. (2012). Trade Barrier Volatility and Agricultural Price Stabilisation. World Dev..

[178] Giordani P.E., Rocha N., Ruta M. (2014). Food Prices and the Multiplier Effect of Trade Policy.

[179] Rude J., An H. (2015). Explaining Grain and Oilseed Price Volatility: The Role of Export Restrictions. Food Policy.

