# Supplementary material for: Impacts of COVID-19 on Trade and Economic Aspects of Food Security: Evidence from 45 Developing Countries
Source: Int J Environ Res Public Health. 2020 Aug 10;17(16):5775. doi: 10.3390/ijerph17165775 (PMC7459461; doi:10.3390/ijerph17165775)
Supplement: Supplementary file 1 [file ijerph-17-05775-s001.pdf]

International Journal of
*Environmental Research and Public Health*
MDPI

**Table S1.** ADF and PP tests results, Group 1.

| Country | Parameters | Y | $X_1$ | $X_2$ | $X_3$ | $X_4$ |
|---|---|---|---|---|---|---|
| Afghanistan | ADF level | −2.68** | −3.72 | −1.98*** | −3.81** | −4.13 |
| | ADF first difference | −3.21** | −3.60* | −3.00** | −2.88*** | −3.85** |
| | PP level | −2.53* | −3.69 | −2.03*** | −3.17** | −4.05* |
| | PP first difference. | −2.67* | −3.44* | −2.76** | −2.74*** | −3.77** |
| Burkina Faso | ADF level | −0.45*** | −2.83** | −0.28* | −2.90** | −4.88*** |
| | ADF first difference | −2.06** | −3.51* | −2.09 | −2.72** | −3.95** |
| | PP level | −0.56*** | −2.44** | −0.62** | −2.85** | −4.69*** |
| | PP first difference. | −2.09** | −3.17* | −2.16 | −2.59** | −3.76** |
| Chad | ADF level | −3.54 | −2.88** | −4.17*** | −2.44 | −3.31 |
| | ADF first difference | −2.86** | −0.74*** | −3.85** | −0.95** | −2.49* |
| | PP level | −3.92 | −2.61** | −4.01*** | −2.18 | −3.17 |
| | PP first difference. | −2.03** | −0.52*** | −3.96** | −0.06** | −2.65* |
| Congo, Democratic Republic | ADF level | −1.05** | −4.13* | −3.33 | −2.97*** | −3.88*** |
| | ADF first difference | −2.55*** | −3.75** | −2.47** | −3.06** | −2.55** |
| | PP level | −1.42** | −4.01* | −3.52* | −2.44*** | −3.53*** |
| | PP first difference. | −2.08*** | −3.59** | −2.04** | −3.18** | −2.47** |
| Ethiopia | ADF level | −3.46** | −1.05*** | −2.74* | −5.66* | −4.86*** |
| | ADF first difference | −2.09*** | −2.15** | −3.06* | −4.99** | −4.05** |
| | PP level | −3.52** | −1.11*** | −2.67* | −5.05* | −4.09*** |
| | PP first difference. | −2.18*** | −2.19** | −2.98* | −4.84** | −3.98** |
| Guinea | ADF level | −3.55* | −3.17 | −4.14** | −3.95*** | −2.19* |
| | ADF first difference | −2.97** | −2.98** | −5.05* | −3.28** | −3.06** |
| | PP level | −3.42* | −3.05 | −4.19** | −3.66*** | −2.35* |
| | PP first difference. | −2.80** | −2.87** | −4.96* | −3.14** | −2.77** |
| Haiti | ADF level | −5.17 | −4.88** | −3.48*** | −2.17** | −0.15** |
| | ADF first difference | −3.19* | −3.10** | −2.87** | −3.42* | −2.34*** |
| | PP level | −4.89 | −4.67** | −3.55*** | −2.53** | −0.32** |
| | PP first difference. | −3.54* | −3.14** | −2.80** | −3.18* | −2.30*** |
| Mali | ADF level | −2.18*** | −2.15*** | −0.88* | −2.16 | −1.85* |
| | ADF first difference | −3.66** | −3.96** | −2.05** | −1.98* | −2.06** |
| | PP level | −2.01*** | −2.12*** | −0.96* | −2.19* | −1.82* |
| | PP first difference. | −3.12** | −3.87** | −2.03** | −1.77* | −2.14** |
| Mozambique | ADF level | −0.97 | −1.54* | −1.09*** | −2.55*** | −1.87 |
| | ADF first difference | −0.45* | −2.67** | −2.14** | −1.98** | −2.09** |
| | PP level | −1.20 | −1.49* | −1.07*** | −2.76*** | −1.23 |
| | PP first difference. | −0.91* | −2.06** | −2.19** | −1.85** | −2.34** |
| Nepal | ADF level | −1.02*** | −0.68** | −1.85* | −2.04* | −1.99* |
| | ADF first difference | −2.28** | −1.84*** | −2.09** | −0.16*** | −1.55** |
| | PP level | −1.16*** | −0.95** | −1.66* | −1.98* | −1.74* |
| | PP first difference. | −2.00** | −1.66*** | −2.13** | −0.85*** | −1.37** |
| Niger | ADF level | −3.18 | −2.63* | −0.86*** | −2.95** | −4.32*** |
| | ADF first difference | −2.76* | −1.77** | −0.75*** | −1.87** | −3.14** |
| | PP level | −2.99* | −2.85* | −1.04*** | −2.86** | −4.07*** |
| | PP first difference. | −2.84* | −2.05*** | −0.69*** | −1.84** | −2.96** |
| Sierra Leone | ADF level | −1.95*** | −4.29* | −1.43* | −2.93*** | −2.88 |
| | ADF first difference | −1.59** | −3.13** | −2.01** | −1.19** | −3.40* |
| | PP level | −2.02*** | −4.18* | −1.72* | −2.75*** | −2.39* |
| | PP first difference. | −1.44** | −3.17** | −2.16** | −1.63** | −3.16* |
| Tajikistan | ADF level | −5.40* | −2.29*** | −1.08* | −0.99* | −1.37** |
| | ADF first difference | −4.43* | −1.86** | −2.53** | −1.85** | −0.69*** |
| | PP level | −4.97* | −3.01** | −1.27* | −0.74* | −1.08** |
| | PP first difference. | −4.50* | −2.35** | −2.59** | −1.90** | −0.70*** |
| Tanzania | ADF level | −0.77*** | −1.02* | −0.55*** | −2.75** | −1.44* |
| | ADF first difference | −1.20** | −1.47** | −1.47** | −1.59** | −2.00** |
| | PP level | −0.93*** | −1.24* | −0.68*** | −2.50** | −1.46* |
| | PP first difference. | −1.34** | −1.56** | −1.39** | −1.77** | −2.09** |
| Yemen | ADF level | −3.46* | −2.55*** | −4.94* | −3.18* | −5.33*** |
| | ADF first difference | −4.28** | −1.48** | −3.85** | −2.57 | −6.50*** |
| | PP level | −3.09* | −2.39*** | −4.28* | −3.03* | −5.47*** |
| | PP first difference. | −4.00** | −1.90** | −3.06** | −2.66 | −6.18*** |

*Y* = number of people with insufficient food consumption; $X_1$ = number of confirmed COVID-19 cases; $X_2$ = balance of food trade; $X_3$ = food inflation; $X_4$ = currency exchange; *,**,*** = significance at 10% level, 5% level, and 1% level, respectively. Source: authors' development.

**Table 2.** ADF and PP tests results, Group 2.

| Country | Parameters | Y | $X_1$ | $X_2$ | $X_3$ | $X_4$ |
|---|---|---|---|---|---|---|
| Bangladesh | ADF level | −3.86** | −2.25*** | −3.93** | −1.49** | −2.06** |
| | ADF first difference | −2.54* | −1.66** | −4.60** | −2.00** | −1.47** |
| | PP level | −3.03** | −1.90*** | −3.66** | −1.37** | −2.58** |
| | PP first difference. | −2.38* | −1.73** | −4.39** | −2.14** | −1.45** |
| Bolivia | ADF level | −0.77*** | −1.54* | −1.78* | −1.12*** | −1.39*** |
| | ADF first difference | −1.05** | −2.62** | −2.04** | −0.98** | −0.86** |
| | PP level | −0.49*** | −1.39* | −1.70* | −1.17*** | −1.27*** |
| | PP first difference. | −1.08** | −2.50** | −1.95** | −0.95** | −0.83** |
| Cambodia | ADF level | −2.84 | −3.06*** | −2.18*** | −2.68* | −2.74* |
| | ADF first difference | −3.03** | −3.19** | −2.76** | −1.00* | −2.59 |
| | PP level | −2.88 | −3.13*** | −2.04*** | −2.55* | −2.70* |
| | PP first difference. | −3.12** | −3.40** | −2.39** | −1.06 | −2.68 |
| Cameroon | ADF level | −2.15*** | −2.02* | −3.14 | −2.75** | −1.84*** |
| | ADF first difference | −1.60** | −1.58** | −2.05** | −1.20** | −2.06** |
| | PP level | −2.19*** | −2.73* | −3.38* | −2.27** | −1.77*** |
| | PP first difference. | −1.54** | −1.42** | −2.19*** | −1.49** | −2.18** |
| Cote d'Ivoire | ADF level | −4.55*** | −3.09*** | −5.10* | −3.86*** | −4.04* |
| | ADF first difference | −3.16** | −3.88** | −4.07** | −2.85** | −3.80** |
| | PP level | −4.13*** | −3.17*** | −6.11** | −3.10*** | −4.17* |
| | PP first difference. | −3.50** | −3.50** | −4.24** | −2.49** | −3.75** |
| India | ADF level | −0.99*** | −1.04*** | −0.67** | −1.22** | −1.73** |
| | ADF first difference | −1.04** | −1.33** | −1.64** | −1.64** | −1.95*** |
| | PP level | −0.75*** | −1.48*** | −0.70** | −1.48** | −1.70** |
| | PP first difference. | −1.28** | −1.59** | −1.53** | −1.72** | −1.52*** |
| Indonesia | ADF level | −2.06* | −3.95* | −2.59*** | −2.90*** | −3.44** |
| | ADF first difference | −2.77** | −2.27* | −3.06** | −3.08** | −4.03** |
| | PP level | −2.15* | −3.90* | −2.63*** | −2.74*** | −3.81** |
| | PP first difference. | −2.90* | −2.02** | −3.11** | −3.16** | −4.08** |
| Kenya | ADF level | −5.12*** | −4.03*** | −6.40* | −5.19 | −5.01* |
| | ADF first difference | −4.40** | −3.17** | −4.13** | −4.05* | −4.55* |
| | PP level | −6.94*** | −3.08*** | −6.93* | −4.88 | −4.96* |
| | PP first difference. | −4.00** | −3.54** | −4.19** | −4.16* | −4.97* |
| Kyrgyzstan | ADF level | −2.14** | −2.05** | −3.17*** | −2.09*** | −2.15*** |
| | ADF first difference | −2.33** | −3.14* | −2.99** | −2.18** | −2.04** |
| | PP level | −2.09** | −1.86** | −3.82*** | −2.66*** | −2.19*** |
| | PP first difference. | −2.85** | −3.18* | −2.70** | −2.47** | −2.08** |
| Nigeria | ADF level | −0.47*** | −1.00*** | −0.98*** | −1.03*** | −0.87*** |
| | ADF first difference | −1.28** | −1.39** | −1.21** | −1.30** | −1.06** |
| | PP level | −0.96*** | −1.21*** | −0.75*** | −1.09*** | −0.95*** |
| | PP first difference. | −1.14** | −1.20** | −1.63** | −1.24** | −1.12** |
| Pakistan | ADF level | −4.44 | −6.02** | −5.92* | −3.00* | −4.83** |
| | ADF first difference | −5.60* | −4.11** | −4.96** | −2.95* | −5.01** |
| | PP level | −4.17* | −5.44** | −4.09** | −3.15** | −4.05** |
| | PP first difference. | −5.05** | −4.08** | −4.00** | −2.88* | −5.14** |
| Philippines | ADF level | −2.17*** | −2.01*** | −2.84* | −3.05** | −2.06** |
| | ADF first difference | −3.03** | −1.45** | −3.07** | −2.57** | −2.55** |
| | PP level | −2.95*** | −2.26*** | −2.93** | −2.86** | −2.19** |
| | PP first difference. | −3.11** | −1.32** | −3.09** | −2.69** | −3.10** |
| Tunisia | ADF level | −6.55* | −5.64* | −6.58*** | −4.11*** | −5.66** |
| | ADF first difference | −5.50** | −6.08** | −4.12* | −5.00** | −4.04** |
| | PP level | −7.12* | −5.98* | −5.26*** | −4.01*** | −4.18** |
| | PP first difference. | −6.02** | −6.15** | −4.19* | −5.17** | −4.09** |
| Vietnam | ADF level | −3.15*** | −2.96*** | −3.44* | −4.04* | −3.55* |
| | ADF first difference | −2.97** | −1.99** | −2.77** | −3.53** | −3.60** |
| | PP level | −3.01*** | −2.80*** | −3.86 | −3.99 | −3.04* |
| | PP first difference. | −2.88** | −1.75** | −2.70** | −3.07** | −3.11** |
| Zambia | ADF level | −1.06 | −1.22** | −1.43*** | −1.50*** | −1.94*** |
| | ADF first difference | −1.95** | −2.07*** | −2.58** | −2.36** | −2.27** |
| | PP level | −1.20* | −1.31** | −1.48*** | −1.67*** | −1.88*** |
| | PP first difference. | −2.02** | −2.16*** | −2.47** | −2.04** | −2.40** |

$Y$ = number of people with insufficient food consumption; $X_1$ = number of confirmed COVID-19 cases; $X_2$ = balance of food trade; $X_3$ = food inflation; $X_4$ = currency exchange; *,**,*** = significance at 10% level, 5% level, and 1% level, respectively. Source: authors' development.

**Table S3.** ADF and PP tests results, Group 3.

| Country | Parameters | Y | $X_1$ | $X_2$ | $X_3$ | $X_4$ |
|---|---|---|---|---|---|---|
| Algeria | ADF level | −0.55*** | −1.24** | −0.98* | −1.35** | −1.01*** |
| | ADF first difference | −1.14*** | −1.32*** | −1.05** | −0.96*** | −1.25** |
| | PP level | −0.63*** | −1.95** | −0.87* | −1.55** | −1.08*** |
| | PP first difference. | −1.30*** | −1.40*** | −1.23** | −0.70*** | −1.34** |
| Botswana | ADF level | −2.00* | −2.35* | −2.44*** | −3.40* | −2.99** |
| | ADF first difference | −2.88** | −2.02** | −3.35** | −2.96** | −3.43* |
| | PP level | −2.17* | −2.19* | −2.85*** | −3.12* | −2.57** |
| | PP first difference. | −2.64** | −2.40** | −3.04** | −2.85** | −3.28* |
| Colombia | ADF level | −5.55** | −4.56*** | −3.58* | −5.63*** | −6.16** |
| | ADF first difference | −4.64** | −3.17** | −4.55** | −4.07** | −5.19** |
| | PP level | −5.03** | −4.14*** | −4.00 | −4.99*** | −5.97** |
| | PP first difference. | −4.88** | −3.95** | −5.06** | −5.15** | −5.08** |
| Dominican Republic | ADF level | −3.13*** | −2.96* | −3.75** | −1.53* | −2.50* |
| | ADF first difference | −2.94** | −3.44** | −2.99** | −2.01** | −3.40** |
| | PP level | −3.05*** | −3.17* | −2.50** | −1.66* | −2.54* |
| | PP first difference. | −2.76** | −3.09** | −3.05** | −2.43** | −2.98** |
| Ecuador | ADF level | −1.55** | −0.78*** | −1.21*** | −0.82*** | −1.76*** |
| | ADF first difference | −2.73*** | −1.22** | −2.07** | −1.34** | −2.02** |
| | PP level | −1.42** | −0.84*** | −1.33*** | −0.70*** | −1.56*** |
| | PP first difference. | −2.84*** | −1.45** | −2.38** | −1.55** | −2.47** |
| Guatemala | ADF level | −0.15 | −0.46* | −1.06* | −1.96** | −1.04* |
| | ADF first difference | −0.90** | −0.67** | −1.22** | −0.95*** | −1.33** |
| | PP level | −0.34 | −0.39** | −1.14* | −1.13** | −1.25* |
| | PP first difference. | −0.88** | −0.40** | −1.35** | −0.88*** | −1.24** |
| Iran | ADF level | −2.15* | −2.94*** | −3.21*** | −1.99** | −2.17** |
| | ADF first difference | −2.19** | −1.95** | −2.76** | −2.06* | −2.00* |
| | PP level | −2.66* | −2.60*** | −2.18*** | −1.90** | −2.45** |
| | PP first difference. | −2.40** | −1.87** | −2.59** | −2.17* | −2.14** |
| Iraq | ADF level | −5.81*** | −4.44*** | −6.06* | −4.75** | −4.18** |
| | ADF first difference | −4.99** | −4.90** | −4.00** | −3.86** | −3.75* |
| | PP level | −5.36*** | −3.58*** | −6.12* | −4.70** | −4.73** |
| | PP first difference. | −4.30** | −4.51** | −4.04** | −3.09** | −3.84* |
| Jordan | ADF level | −1.15* | −2.53* | −1.08** | −2.39* | −1.90** |
| | ADF first difference | −2.00** | −1.97** | −1.45** | −1.90*** | −1.47* |
| | PP level | −1.10 | −2.92 | −1.26** | −2.44** | −1.83** |
| | PP first difference. | −2.05** | −1.83** | −1.67** | −1.99** | −1.55* |
| Lebanon | ADF level | −3.07*** | −2.59*** | −2.06*** | −3.05* | −2.18*** |
| | ADF first difference | −2.69** | −2.41** | −1.95** | −2.97** | −2.42** |
| | PP level | −2.86*** | −2.17*** | −2.94*** | −3.44** | −2.36*** |
| | PP first difference. | −2.50** | −2.60** | −1.50** | −2.40** | −2.59** |
| Libya | ADF level | −3.28* | −3.33 | −3.19 | −5.15*** | −4.44* |
| | ADF first difference | −2.99** | −4.21* | −2.28** | −4.40** | −3.60** |
| | PP level | −3.20* | −3.45* | −3.04* | −5.83*** | −4.03** |
| | PP first difference. | −2.54** | −4.02* | −2.36** | −4.35** | −3.56** |
| Namibia | ADF level | −0.66*** | −1.05*** | −0.98* | −1.26* | −0.83* |
| | ADF first difference | −0.75** | −0.64** | −1.11** | −2.05** | −1.17** |
| | PP level | −0.84*** | −1.23*** | −0.54** | −1.29** | −0.44* |
| | PP first difference. | −0.39** | −0.95** | −1.19** | −2.00** | −1.29** |
| Peru | ADF level | −1.25 | −1.60* | −1.43*** | −1.50* | −1.66*** |
| | ADF first difference | −2.06* | −2.83** | −2.04** | −0.67** | −0.95** |
| | PP level | −1.28* | −1.55** | −1.86*** | −1.42* | −1.74*** |
| | PP first difference. | −2.14* | −2.40** | −2.00** | −0.71** | −0.93** |
| Sri Lanka | ADF level | −6.33*** | −4.12 | −7.18* | −5.45*** | −6.13* |
| | ADF first difference | −4.74** | −5.38* | −6.15** | −3.18** | −5.83** |
| | PP level | −5.90** | −5.06* | −6.39* | −4.86*** | −5.48** |
| | PP first difference. | −4.55** | −4.19** | −5.06** | −3.44** | −5.30** |
| Turkey | ADF level | −2.48*** | −1.70*** | −2.07*** | −1.74* | −2.54** |
| | ADF first difference | −3.13** | −2.15** | −2.94** | −2.01** | −2.15*** |
| | PP level | −1.49*** | −1.99*** | −2.15*** | −1.85* | −2.73** |
| | PP first difference. | −3.04** | −2.50** | −2.80** | −2.15** | −2.60*** |

$Y$ = number of people with insufficient food consumption; $X_1$ = number of confirmed COVID-19 cases; $X_2$ = balance of food trade; $X_3$ = food inflation; $X_4$ = currency exchange; *,**,*** = significance at 10% level, 5% level, and 1% level, respectively. Source: authors' development.

**Table S4.** ARDL short-run estimates, Group 1.

| Country | Parameter | $\Delta X_1$ | $\Delta X_2$ | $\Delta X_3$ | $\Delta X_4$ | ECM |
|---|---|---|---|---|---|---|
| Afghanistan | Coefficient | 0.0349 | −0.1512 | 0.1270 | 0.0145 | −0.2021 |
| | t-stat | 2.1275 | −3.1855 | 2.1187 | 3.7719 | −3.4062 |
| | Prob | 0.0116** | 0.0184** | 0.5048** | 0.4034* | 0.0001* |
| Burkina Faso | Coefficient | −0.1371 | −0.4077 | 0.5284 | 0.1293 | −0.3003 |
| | t-stat | 1.9773 | 2.1274 | 3.2876 | 2.8821 | −1.9920 |
| | Prob | 0.0370* | 0.0993* | 0.1882*** | 0.0818** | 0.0000** |
| Chad | Coefficient | −0.0152 | −0.0812 | 0.1194 | −0.4443 | 0.7219 |
| | t-stat | 2.4138 | 3.2296 | −2.1895 | 2.2068 | 2.1726 |
| | Prob | 0.0487*** | 0.1828*** | 0.0332** | 0.0481* | 0.0014** |
| Congo, Democratic Republic | Coefficient | 0.1277 | 0.0319 | 0.2910 | 0.0835 | −0.5592 |
| | t-stat | 1.4005 | 5.2964 | 7.1295 | 4.8023 | −1.3443 |
| | Prob | 0.0588* | 0.1627*** | 0.0285** | 0.0994** | 0.0010*** |
| Ethiopia | Coefficient | −0.0313 | 0.0299 | 0.3063 | −0.9500 | −0.0377 |
| | t-stat | −4.8291 | −2.3953 | −5.0882 | 4.2910 | −1.7738 |
| | Prob | 0.0472** | 0.1006** | 0.0385*** | 0.1333* | 0.0044* |
| Guinea | Coefficient | 0.15721 | 0.0555 | 0.4065 | 0.0913 | −0.7954 |
| | t-stat | 3.1180 | 7.0377 | 3.1196 | 1.8032 | −1.1749 |
| | Prob | 0.0187** | 0.1978*** | 0.0582*** | 0.3966*** | 0.0153*** |
| Haiti | Coefficient | −0.1148 | 0.2554 | 0.1491 | 0.3083 | 0.5309 |
| | t-stat | 4.1599 | 3.7043 | −1.1937 | 3.2284 | 1.0526 |
| | Prob | 0.1862*** | 0.4390*** | 0.3915*** | 0.0996*** | 0.0007** |
| Mali | Coefficient | 0.1334 | −0.1799 | 0.4237 | −0.0885 | −0.4930 |
| | t-stat | −1.1680 | 4.9382 | 2.4920 | 8.1863 | −1.1285 |
| | Prob | 0.0828* | 0.0054** | 0.0455** | 0.0499* | 0.0004* |
| Mozambique | Coefficient | 0.0482 | 0.2649 | 0.8003 | 0.3915 | 0.2064 |
| | t-stat | 2.1154 | −2.4933 | 4.1801 | −4.2901 | 2.1192 |
| | Prob | 0.0933*** | 0.0491** | 0.3338*** | 0.0522*** | 0.0046*** |
| Nepal | Coefficient | −0.0338 | 0.0174 | 0.1904 | 0.0505 | −0.1883 |
| | t-stat | 5.3991 | 5.3910 | −2.8302 | 6.1000 | −2.1142 |
| | Prob | 0.0884*** | 0.0291*** | 0.2988** | 0.3919** | 0.0055** |
| Niger | Coefficient | 0.1773 | −0.0506 | 0.2159 | 0.2703 | −0.2390 |
| | t-stat | 2.2406 | 7.2593 | 4.3491 | −2.5099 | −2.7048 |
| | Prob | 0.5321** | 0.0488* | 0.0039* | 0.0487** | 0.0030*** |
| Sierra Leone | Coefficient | 0.0538 | 0.7401 | 0.5593 | −0.5698 | 0.5554 |
| | t-stat | 4.3900 | 4.4389 | 3.2115 | 6.7302 | 1.1193 |
| | Prob | 0.2291*** | 0.0883** | 0.0599*** | 0.1776** | 0.0019* |
| Tajikistan | Coefficient | 0.1399 | 0.5450 | 0.7126 | 0.5520 | −0.1984 |
| | t-stat | 6.1002 | 5.1991 | 3.4553 | 3.4098 | −2.3250 |
| | Prob | 0.0058*** | 0.0046*** | 0.2964*** | 0.1865*** | 0.0009*** |
| Tanzania | Coefficient | 0.1391 | −0.2553 | 0.0203 | −0.4609 | −0.5072 |
| | t-stat | 1.1443 | −1.1905 | −7.1392 | 6.2004 | −3.6084 |
| | Prob | 0.0993** | 0.1008* | 0.0355* | 0.0830* | 0.0014** |
| Yemen | Coefficient | −0.0309 | 0.6921 | 0.6665 | 0.0195 | −0.1988 |
| | t-stat | −2.1866 | 3.2077 | 2.5043 | −5.1006 | −2.5829 |
| | Prob | 0.0554* | 0.0973*** | 0.1115*** | 0.1863*** | 0.0110*** |

$X_1$ = number of confirmed COVID-19 cases; $X_2$ = balance of food trade; $X_3$ = food inflation; $X_4$ = currency exchange; *,**,*** = significance at 10% level, 5% level, and 1% level, respectively. Source: authors’ development.

**Table S5.** ARDL short-run estimates, Group 2.

| Country | Parameter | ΔX1 | ΔX2 | ΔX3 | ΔX4 | ECM |
|---|---|---|---|---|---|---|
| Bangladesh | Coefficient | 0.3599 | 0.0582 | 0.1103 | 0.0861 | 0.1472 |
| | t-stat | −3.8921 | −1.8298 | 3.2918 | 1.0284 | 1.5590 |
| | Prob | 0.0482** | 0.1985*** | 0.0943*** | 0.0071*** | 0.0487** |
| Bolivia | Coefficient | 0.5018 | 0.0345 | 0.0337 | 0.0046 | 0.1876 |
| | t-stat | 7.1924 | −2.8882 | 4.4041 | 2.2965 | 3.1209 |
| | Prob | 0.0386* | 0.0496*** | 0.0182*** | 0.1283*** | 0.0938*** |
| Cambodia | Coefficient | −0.0664 | −0.5001 | −0.4771 | −0.0966 | −0.4527 |
| | t-stat | −3.3836 | 5.7830 | 3.6019 | 1.4498 | −1.8403 |
| | Prob | 0.0485*** | 0.2785** | 0.3918* | 0.0835*** | 0.0829** |
| Cameroon | Coefficient | 0.1190 | 0.1834 | 0.1493 | 0.0491 | 0.3875 |
| | t-stat | 5.3903 | −3.3439 | −1.4811 | 1.0073 | 3.4912 |
| | Prob | 0.1388*** | 0.0594** | 0.0083** | 0.0689*** | 0.0015*** |
| Cote d’Ivoire | Coefficient | 0.0690 | 0.0841 | 0.3550 | 0.2094 | −0.3187 |
| | t-stat | 2.2702 | 4.9964 | 2.4387 | −4.3309 | −3.1030 |
| | Prob | 0.0559*** | 0.1285*** | 0.0472*** | 0.5018** | 0.0593** |
| India | Coefficient | 0.5320 | 0.3401 | −0.0400 | 0.3343 | −0.2981 |
| | t-stat | 7.5182 | 6.0003 | −6.0909 | −3.1286 | −3.0844 |
| | Prob | 0.0491*** | 0.0991*** | 0.0424*** | 0.0488** | 0.0011*** |
| Indonesia | Coefficient | 0.3476 | 0.2918 | −0.0309 | 0.5862 | 0.4299 |
| | t-stat | 3.2777 | 5.4391 | −3.9285 | −3.1174 | 1.9392 |
| | Prob | 0.0593** | 0.1922*** | 0.1800*** | 0.0015** | 0.0006* |
| Kenya | Coefficient | 0.2375 | 0.4291 | 0.2959 | 0.4006 | 0.3918 |
| | t-stat | −2.0086 | −2.1314 | 4.2072 | 2.2099 | 2.1773 |
| | Prob | 0.0059* | 0.0345*** | 0.1288** | 0.2916*** | 0.0110** |
| Kyrgyzstan | Coefficient | 0.0505 | 0.1096 | 0.3829 | 0.3096 | −0.1559 |
| | t-stat | −4.4134 | 2.0055 | 6.4012 | 3.5482 | −2.0672 |
| | Prob | 0.1729*** | 0.3921** | 0.3023*** | 0.0926** | 0.0018*** |
| Nigeria | Coefficient | 0.1006 | 0.1128 | 0.4472 | 0.4814 | 0.2850 |
| | t-stat | 5.0399 | −1.5039 | 1.5286 | 6.4088 | 3.1195 |
| | Prob | 0.0098** | 0.5920** | 0.0053*** | 0.1892* | 0.0055** |
| Pakistan | Coefficient | 0.5593 | 0.2918 | −0.4925 | 0.8863 | 0.2917 |
| | t-stat | 2.0546 | 2.5503 | 4.0938 | 6.0582 | 3.2001 |
| | Prob | 0.0059*** | 0.0920*** | 0.4553** | 0.0076* | 0.0085*** |
| Philippines | Coefficient | 0.8265 | 0.4439 | 0.1097 | 0.1294 | −0.2983 |
| | t-stat | 1.4924 | −1.0965 | 1.2876 | 2.4006 | −2.0945 |
| | Prob | 0.0827* | 0.0592** | 0.4485* | 0.3193** | 0.0096** |
| Tunisia | Coefficient | 0.1285 | 0.3900 | 0.2157 | 0.1884 | 0.4285 |
| | t-stat | −2.7038 | 1.3886 | 2.7729 | 2.1009 | 2.1006 |
| | Prob | 0.0487** | 0.0093** | 0.0900** | 0.4981*** | 0.0007* |
| Vietnam | Coefficient | −0.3909 | 0.0184 | 0.3051 | 0.2286 | −0.0783 |
| | t-stat | 3.7544 | 3.8916 | −2.0044 | 1.5390 | −1.9504 |
| | Prob | 0.0593*** | 0.0996*** | 0.0093** | 0.0135** | 0.0167** |
| Zambia | Coefficient | −0.0138 | 0.5197 | 0.6290 | −0.4499 | 0.3901 |
| | t-stat | 1.2884 | 4.0091 | 2.6664 | −6.2030 | 1.8026 |
| | Prob | 0.0927*** | 0.1952* | 0.0844** | 0.0086* | 0.0095** |

$X_1$ = number of confirmed COVID-19 cases; $X_2$ = balance of food trade; $X_3$ = food inflation; $X_4$ = currency exchange; *,**,*** = significance at 10% level, 5% level, and 1% level, respectively. Source: authors’ development.

**Table S6.** ARDL short-run estimates, Group 3.

| Country | Parameter | $\Delta X_1$ | $\Delta X_2$ | $\Delta X_3$ | $\Delta X_4$ | ECM |
|---|---|---|---|---|---|---|
| Algeria | Coefficient | 0.0559 | 0.6302 | −0.7728 | 0.1954 | −0.3309 |
| | t-stat | 2.5443 | 1.1095 | −1.2887 | −1.2099 | −1.2875 |
| | Prob | 0.0421* | 0.0598** | 0.0042* | 0.0586** | 0.0048*** |
| Botswana | Coefficient | −0.4097 | 0.4154 | 0.0499 | 0.0071 | 0.3977 |
| | t-stat | 3.0854 | −1.0773 | 2.0947 | −1.8382 | 3.2114 |
| | Prob | 0.0095* | 0.0883*** | 0.0994** | 0.2186** | 0.0093** |
| Colombia | Coefficient | 0.0392 | 0.4928 | 0.1630 | 0.0449 | 0.1074 |
| | t-stat | 2.4981 | 2.0392 | 3.1114 | 2.4985 | 2.3029 |
| | Prob | 0.0056*** | 0.0185*** | 0.0486** | 0.1987*** | 0.0027*** |
| Dominican Republic | Coefficient | 0.0882 | 0.3096 | −0.1719 | 0.1285 | −0.3028 |
| | t-stat | 2.4895 | 3.1175 | 2.1332 | 2.8850 | −3.3926 |
| | Prob | 0.0977*** | 0.1194*** | 0.0090*** | 0.0187*** | 0.0029** |
| Ecuador | Coefficient | 0.4022 | 0.1135 | 0.2389 | 0.5096 | 0.1975 |
| | t-stat | 7.0974 | 6.2038 | −2.5550 | −2.3918 | 1.8443 |
| | Prob | 0.0885*** | 0.2876** | 0.0049*** | 0.0111** | 0.0138* |
| Guatemala | Coefficient | 0.1791 | 0.1990 | −0.3388 | 0.1120 | 0.4983 |
| | t-stat | 4.7367 | 4.3009 | −1.8727 | 3.2378 | 2.0987 |
| | Prob | 0.0059** | 0.0177** | 0.1284* | 0.0295* | 0.0364** |
| Iran | Coefficient | −0.0509 | −0.1845 | −0.4003 | −0.0862 | 0.2678 |
| | t-stat | 2.1986 | 1.2209 | −5.1767 | 2.8475 | 3.2784 |
| | Prob | 0.0975* | 0.1878* | 0.0340** | 0.2901* | 0.0502** |
| Iraq | Coefficient | 0.0482 | 0.1190 | −0.9498 | 0.1376 | −0.4985 |
| | t-stat | −3.8863 | 2.0493 | −2.1447 | 1.8309 | −1.3873 |
| | Prob | 0.0985** | 0.0053** | 0.1985* | 0.0043** | 0.0387** |
| Jordan | Coefficient | −0.1185 | 0.7392 | 0.0399 | 0.4008 | −0.2254 |
| | t-stat | 3.5007 | 3.8299 | 3.0404 | −5.1223 | −2.0980 |
| | Prob | 0.0444* | 0.0083*** | 0.0028** | 0.1174*** | 0.0127*** |
| Lebanon | Coefficient | 0.1761 | 0.5042 | 0.4094 | 0.2886 | −0.3104 |
| | t-stat | 2.4332 | 4.0939 | 4.0125 | −3.7748 | −4.0638 |
| | Prob | 0.0054** | 0.0118*** | 0.0137** | 0.2864*** | 0.0019** |
| Libya | Coefficient | −0.2006 | 0.3440 | 0.1876 | 0.1555 | 0.1986 |
| | t-stat | 7.0287 | −3.2264 | 3.9275 | 2.2890 | 3.1183 |
| | Prob | 0.0195** | 0.0870*** | 0.0089*** | 0.0482*** | 0.0054* |
| Namibia | Coefficient | −0.5016 | 0.2928 | 0.1231 | 0.3968 | −0.2359 |
| | t-stat | −2.4729 | 3.4433 | −1.3918 | 2.5520 | −1.2263 |
| | Prob | 0.0192* | 0.0042*** | 0.0303** | 0.2666*** | 0.0149** |
| Peru | Coefficient | 0.5441 | 0.2856 | 0.0036 | 0.0465 | 0.0585 |
| | t-stat | 3.0665 | 5.3771 | 2.1098 | −2.7743 | 1.1972 |
| | Prob | 0.0490*** | 0.2885** | 0.1974* | 0.0283* | 0.0013*** |
| Sri Lanka | Coefficient | 0.3352 | −0.2549 | 0.5043 | −0.1665 | 0.0064 |
| | t-stat | −1.7776 | 1.3981 | 3.0932 | 6.0554 | 3.0336 |
| | Prob | 0.0554** | 0.0043* | 0.0012* | 0.0590** | 0.0222** |
| Turkey | Coefficient | 0.0743 | 0.0909 | 0.3553 | 0.0170 | −0.3984 |
| | t-stat | 4.1739 | 3.2030 | −2.1126 | −2.0337 | −3.8711 |
| | Prob | 0.0146* | 0.0487* | 0.0388** | 0.0445*** | 0.0550*** |

$X_1$ = number of confirmed COVID-19 cases; $X_2$ = balance of food trade; $X_3$ = food inflation; $X_4$ = currency exchange; *,**,*** = significance at 10% level, 5% level, and 1% level, respectively. Source: authors' development.

**Table S7.** FMOLS and DOLS tests results and ARDL long-run estimates, Group 1.

| Country | Parameters | $X_1$ | $X_2$ | $X_3$ | $X_4$ | Constant |
|---|---|---|---|---|---|---|
| Afghanistan | ARDL coefficient | 0.0285 | −0.1729 | 0.1284 | 0.0183 | −4.8699 |
| | ARDL t-stat | 3.1829** | −4.1294** | 3.9012** | 4.2019* | −2.1845 |
| | FMOLS coefficient | 0.0318 | −0.1782 | 0.2015 | 0.1836 | −3.5097 |
| | FMOLS t-stat | 2.9102** | −4.8472** | 2.4964* | 3.2087* | −2.0836 |
| | DOLS coefficient | 0.0279 | −0.1543 | 0.1711 | 0.1104 | −4.5192 |
| | DOLS t-stat | 3.1991** | −4.0564** | 3.8042** | 4.2192* | −2.1739 |
| Burkina Faso | ARDL coefficient | −0.1128 | −0.3981 | 0.5192 | 0.1038 | −5.1938 |
| | ARDL t-stat | 1.9942* | 2.0994* | 4.0006*** | 1.1022** | −1.5002 |
| | FMOLS coefficient | −0.1764 | −0.3927 | 0.5928 | 0.1602 | −3.9772 |
| | FMOLS t-stat | 1.8974** | 2.2297* | 2.5934** | 1.4997** | −2.9598 |
| | DOLS coefficient | −0.1319 | −0.5051 | 0.6189 | 0.1591 | −4.1683 |
| | DOLS t-stat | 2.1975* | 2.1925* | 2.1985** | 1.1865** | −1.6187 |
| Chad | ARDL coefficient | −0.0185 | −0.0943 | 0.1067 | −0.4902 | 1.6920 |
| | ARDL t-stat | 2.4019*** | 3.1762*** | −3.9058** | 3.1987* | 2.6825 |
| | FMOLS coefficient | −0.1208 | −0.3111 | 0.1529 | −0.3988 | 1.9882 |
| | FMOLS t-stat | 1.5997*** | 1.9772** | −3.5920** | 3.0063* | 3.1592 |
| | DOLS coefficient | −0.1182 | −0.2875 | 0.1288 | −0.4902 | 1.7647 |
| | DOLS t-stat | 2.5817*** | 2.1953** | −3.9291** | 3.1987* | 2.7291 |
| Congo, Democratic Republic | ARDL coefficient | 0.1099 | 0.0211 | 0.1392 | 0.0812 | −4.4892 |
| | ARDL t-stat | 1.3902* | 5.3677*** | 7.0218** | 4.7391** | −1.3827 |
| | FMOLS coefficient | 0.1197 | 0.1298 | 0.2919 | 0.1397 | −2.6999 |
| | FMOLS t-stat | 1.8472* | 4.9287** | 6.0984** | 4.6920** | −2.4773 |
| | DOLS coefficient | 0.1099 | 0.0371 | 0.1997 | 0.1138 | −4.0053 |
| | DOLS t-stat | 1.3904* | 5.4873*** | 7.2815** | 4.5272** | −1.0957 |
| Ethiopia | ARDL coefficient | −0.0281 | 0.0172 | 0.2385 | −0.8491 | −5.0092 |
| | ARDL t-stat | −5.1220** | −3.1492** | −6.9258*** | 5.1299* | −2.6935 |
| | FMOLS coefficient | −0.0492 | 0.0776 | 0.3058 | −0.7991 | −5.9711 |
| | FMOLS t-stat | −2.5908*** | −3.0988** | −4.8989*** | 5.0029* | −2.9876 |
| | DOLS coefficient | −0.0328 | 0.0187 | 0.2865 | −0.8290 | −5.0984 |
| | DOLS t-stat | −4.993** | −3.8772** | −5.2988*** | 5.4832* | −2.0388 |
| Guinea | ARDL coefficient | 0.1741 | 0.0482 | 0.3212 | 0.0817 | −4.8888 |
| | ARDL t-stat | 3.0054** | 7.0011*** | 4.1029*** | 1.7629*** | −1.1457 |
| | FMOLS coefficient | 0.1755 | 0.0692 | 0.4927 | 0.1690 | −4.9921 |
| | FMOLS t-stat | 2.9832** | 4.0982*** | 3.9952** | 2.8892** | −1.2780 |
| | DOLS coefficient | 0.1835 | 0.0467 | 0.5197 | 0.1518 | −4.8726 |
| | DOLS t-stat | 2.5672** | 5.1382*** | 4.1593** | 2.3082** | −1.1550 |
| Haiti | ARDL coefficient | −0.0995 | 0.2918 | 0.1392 | 0.4987 | 2.5683 |
| | ARDL t-stat | 4.1972*** | 3.9019*** | −1.0289*** | 3.1987*** | 1.0046 |
| | FMOLS coefficient | −0.1297 | 0.2887 | 0.1289 | 0.5198 | 2.5298 |
| | FMOLS t-stat | 3.9077*** | 3.0983** | −1.5928*** | 2.5929** | 2.1395 |
| | DOLS coefficient | −0.1198 | 0.2278 | 0.1418 | 0.5029 | 2.6589 |
| | DOLS t-stat | 4.2901*** | 2.1995** | −1.3787*** | 2.8859** | 2.0853 |
| Mali | ARDL coefficient | 0.1117 | −0.1284 | 0.4020 | −0.0928 | −3.5833 |
| | ARDL t-stat | −1.1213* | 5.2812** | 3.1718** | 9.2918* | −1.0425 |
| | FMOLS coefficient | 0.1290 | −0.1897 | 0.5902 | −0.1887 | −2.0873 |
| | FMOLS t-stat | −1.3887* | 3.9084** | 3.9895* | 6.8983* | −1.0822 |
| | DOLS coefficient | 0.1489 | −0.1392 | 0.4108 | −0.1798 | −3.5787 |
| | DOLS t-stat | −1.1519* | 4.2294** | 2.9874** | 8.3982* | −1.1182 |
| Mozambique | ARDL coefficient | 0.0014 | 0.2018 | 0.8624 | 0.3890 | 4.1492 |
| | ARDL t-stat | 2.0475*** | −2.4094** | 5.1902*** | −5.111*** | 2.0948 |
| | FMOLS coefficient | 0.1192 | 0.1189 | 0.6652 | 0.3882 | 2.0985 |
| | FMOLS t-stat | 3.2877** | 2.0935*** | 4.9887*** | −3.9872*** | 1.5983 |
| | DOLS coefficient | 0.0298 | 0.0982 | 0.8787 | 0.3386 | 3.1589 |
| | DOLS t-stat | 3.7795** | 3.5972*** | 5.5983*** | −4.6903*** | 2.4123 |
| Nepal | ARDL coefficient | −0.0281 | 0.0041 | 0.1825 | 0.0488 | −5.1947 |
| | ARDL t-stat | 6.3018*** | 4.1975*** | −2.8977** | 6.1998** | −2.3595 |
| | FMOLS coefficient | −0.1490 | 0.0275 | 0.1826 | 0.0712 | −5.0002 |
| | FMOLS t-stat | 2.9955** | 4.0002** | −2.6981** | 5.9921** | −2.5099 |
| | DOLS coefficient | −0.1302 | 0.0487 | 0.1792 | 0.0771 | −5.3028 |
| | DOLS t-stat | 3.5083** | 3.8975** | −2.6778** | 5.2915** | −2.4412 |
| Niger | ARDL coefficient | 0.1662 | −0.0382 | 0.2017 | 0.2900 | −6.2945 |
| | ARDL t-stat | 2.2209** | 8.1924* | 5.2975* | −2.5002** | −3.6800 |
| | FMOLS coefficient | 0.1682 | −0.2184 | 0.2804 | 0.3398 | −5.1284 |

| | | | | | | |
|---|---|---|---|---|---|---|
| | FMOLS t-stat | 1.9925** | 2.0582** | 4.8824* | −2.9882** | −3.1898 |
| | DOLS coefficient | 0.1730 | −0.1985 | 0.2918 | 0.2918 | −5.7287 |
| | DOLS t-stat | 2.3780** | 2.5987** | 5.0006* | −2.8924** | −3.8824 |
| Sierra Leone | ARDL coefficient | 0.0948 | 0.6281 | 0.6927 | −0.5902 | 2.5038 |
| | ARDL t-stat | 5.3810*** | 5.5278** | 4.2019*** | 6.8763** | 1.0002 |
| | FMOLS coefficient | 0.1822 | 0.5122 | 0.4894 | −0.3982 | 2.0032 |
| | FMOLS t-stat | 4.1989** | 4.0828** | 3.5019** | 5.0285** | 1.6729 |
| | DOLS coefficient | 0.1759 | 0.6991 | 0.5284 | −0.4828 | 1.9943 |
| | DOLS t-stat | 3.9981** | 5.0376** | 3.0107** | 5.2787** | 1.8811 |
| Tajikistan | ARDL coefficient | 0.1114 | 0.5393 | 0.7029 | 0.6592 | −3.0592 |
| | ARDL t-stat | 8.1988*** | 5.1782*** | 3.1314*** | 3.4918*** | −2.3098 |
| | FMOLS coefficient | 0.1421 | 0.4019 | 0.5872 | 0.5628 | −2.5882 |
| | FMOLS t-stat | 6.0982*** | 3.0066*** | 3.0881*** | 2.7299** | −2.0021 |
| | DOLS coefficient | 0.1298 | 0.4280 | 0.6938 | 0.5039 | −2.7938 |
| | DOLS t-stat | 7.2875*** | 4.8982*** | 3.1528*** | 2.5982** | −1.9992 |
| Tanzania | ARDL coefficient | 0.1286 | −0.2919 | 0.0176 | −0.4718 | −5.4957 |
| | ARDL t-stat | 1.1904** | −1.1830* | −8.2918* | 9.1987* | −3.5956 |
| | FMOLS coefficient | 0.1392 | −0.4113 | 0.0518 | −0.6024 | −4.0997 |
| | FMOLS t-stat | 1.3020** | −1.2996* | −6.4202* | 6.8993* | −3.9980 |
| | DOLS coefficient | 0.1389 | −0.3092 | 0.0188 | −0.5982 | −4.9821 |
| | DOLS t-stat | 1.2994** | −1.1682* | −6.8821* | 7.1110* | −3.3336 |
| Yemen | ARDL coefficient | −0.0212 | 0.7720 | 0.8021 | 0.0028 | −4.2095 |
| | ARDL t-stat | −3.4881* | 3.9901*** | 2.9413*** | −6.1862*** | −2.6083 |
| | FMOLS coefficient | −0.0441 | 0.6600 | 0.7402 | 0.0202 | −4.0024 |
| | FMOLS t-stat | −3.0022* | 4.2019** | 3.3193*** | −5.0013*** | −3.1497 |
| | DOLS coefficient | −0.0384 | 0.7351 | 0.7744 | 0.0198 | −4.2392 |
| | DOLS t-stat | −3.6920* | 4.5992** | 3.5928*** | −5.7724*** | −3.5590 |

$X_1$ = number of confirmed COVID-19 cases; $X_2$ = balance of food trade; $X_3$ = food inflation; $X_4$ = currency exchange; *,**,*** = significance at 10% level, 5% level, and 1% level, respectively. Source: authors' development.

**Table S8.** FMOLS and DOLS tests results and ARDL long-run estimates, Group 2.

| Country | Parameters | $X_1$ | $X_2$ | $X_3$ | $X_4$ | Constant |
|---|---|---|---|---|---|---|
| Bangladesh | ARDL coefficient | 0.3828 | 0.0482 | 0.0993 | 0.0156 | 2.4735 |
| | ARDL t-stat | −3.8200** | −1.3928*** | 3.6928*** | 1.0054*** | 1.6732 |
| | FMOLS coefficient | 0.4001 | 0.1993 | 0.1309 | 0.0397 | 2.3392 |
| | FMOLS t-stat | −2.0904** | −1.0035** | 2.0686*** | 1.5904** | 1.8883 |
| | DOLS coefficient | 0.3590 | 0.0598 | 0.1192 | 0.0200 | 2.5991 |
| | DOLS t-stat | −3.4983** | −1.3522*** | 3.2094*** | 1.0402*** | 1.6529 |
| Bolivia | ARDL coefficient | 0.4913 | 0.0201 | 0.0294 | 0.0182 | 4.8209 |
| | ARDL t-stat | 9.2066* | −2.9483*** | 4.2877*** | 2.1985*** | 3.2555 |
| | FMOLS coefficient | 0.5921 | 0.0628 | 0.0582 | 0.0778 | 3.5026 |
| | FMOLS t-stat | 7.9093** | −2.9940** | 3.9002*** | 1.8294*** | 3.0053 |
| | DOLS coefficient | 0.5199 | 0.0494 | 0.0400 | 0.0699 | 3.7844 |
| | DOLS t-stat | 8.3192* | −2.6850*** | 3.4995*** | 1.7877*** | 3.1920 |
| Cambodia | ARDL coefficient | −0.0583 | −0.4877 | −0.4899 | −0.0831 | −2.4671 |
| | ARDL t-stat | −4.3921*** | 5.7092** | 3.6948* | 1.5937*** | −1.9932 |
| | FMOLS coefficient | −0.0992 | −0.6043 | −0.7277 | −0.0891 | −2.0694 |
| | FMOLS t-stat | −2.0055** | 5.1984** | 2.5593** | 1.9924*** | −1.9902 |
| | DOLS coefficient | −0.0691 | −0.5920 | −0.5921 | −0.0782 | −2.5584 |
| | DOLS t-stat | −3.7882*** | 4.8923** | 3.5566* | 1.7085*** | −2.0043 |
| Cameroon | ARDL coefficient | 0.0372 | 0.1752 | 0.0948 | 0.0927 | 6.2987 |
| | ARDL t-stat | 5.3928*** | −4.3991** | −1.2583** | 1.5274*** | 4.2076 |
| | FMOLS coefficient | 0.1193 | 0.2190 | 0.1094 | 0.1006 | 5.0909 |
| | FMOLS t-stat | 3.0026** | −3.0924** | −1.2283** | 1.4928*** | 3.8892 |
| | DOLS coefficient | 0.0829 | 0.1956 | 0.0952 | 0.0994 | 5.8890 |
| | DOLS t-stat | 4.6728*** | −4.0482** | −1.1196** | 1.5671*** | 4.9977 |
| Cote d'Ivoire | ARDL coefficient | 0.0483 | 0.0728 | 0.3295 | 0.1928 | −5.2696 |
| | ARDL t-stat | 4.2916*** | 5.2985*** | 2.1957*** | −6.2918** | −4.1182 |
| | FMOLS coefficient | 0.0890 | 0.0983 | 0.4287 | 0.1906 | −5.0434 |
| | FMOLS t-stat | 4.0915*** | 4.9289*** | 2.2286*** | 5.8921** | −4.8985 |
| | DOLS coefficient | 0.0666 | 0.0834 | 0.3488 | 0.1899 | −5.5529 |
| | DOLS t-stat | 4.8921*** | 4.8743*** | 2.1684*** | −6.1400** | −4.1592 |
| India | ARDL coefficient | 0.4820 | 0.3863 | −0.0823 | 0.2986 | −5.1903 |
| | ARDL t-stat | 9.2914*** | 7.2917*** | −9.4182*** | −4.2975** | −4.0002 |
| | FMOLS coefficient | 0.4029 | 0.3892 | −0.0787 | 0.3281 | −4.9889 |
| | FMOLS t-stat | 8.2014*** | 6.5982*** | −8.4985*** | −4.9990** | −4.5024 |
| | DOLS coefficient | 0.4584 | 0.3771 | −0.0988 | 0.3044 | −5.2993 |
| | DOLS t-stat | 8.0778*** | 6.3335*** | −8.6683*** | −4.5838** | −4.8195 |
| Indonesia | ARDL coefficient | 0.2285 | 0.2227 | −0.0721 | 0.5724 | 2.1790 |
| | ARDL t-stat | 4.2985** | 4.5928*** | −4.9289*** | −3.2983** | 1.9624 |
| | FMOLS coefficient | 0.3054 | 0.2309 | −0.0672 | 0.5548 | 2.3881 |
| | FMOLS t-stat | 4.5220** | 4.6083*** | −4.6718*** | −2.9896** | 1.9999 |
| | DOLS coefficient | 0.2386 | 0.2254 | −0.0680 | 0.5655 | 2.1666 |
| | DOLS t-stat | 4.0631** | 4.5992*** | −4.8843*** | −3.0633** | 1.8078 |
| Kenya | ARDL coefficient | 0.2010 | 0.4999 | 0.2784 | 0.4443 | 3.2755 |
| | ARDL t-stat | −3.5917* | −3.0288*** | 5.2903** | 2.1985*** | 3.0585 |
| | FMOLS coefficient | 0.2286 | 0.4492 | 0.3392 | 0.5027 | 3.3386 |
| | FMOLS t-stat | −2.9018* | −1.6987*** | 4.6029** | 1.7692*** | 3.1295 |
| | DOLS coefficient | 0.2133 | 0.4973 | 0.2598 | 0.4559 | 3.2853 |
| | DOLS t-stat | −3.6005* | −2.0682*** | 4.5902** | 1.8843*** | 3.1000 |
| Kyrgyzstan | ARDL coefficient | 0.0392 | 0.1562 | 0.6999 | 0.3892 | −4.1446 |
| | ARDL t-stat | −5.3992*** | 3.1778** | 7.2808*** | 3.3816** | −3.9836 |
| | FMOLS coefficient | 0.0596 | 0.1567 | 0.7185 | 0.4399 | −4.3921 |
| | FMOLS t-stat | −4.9928*** | 2.9830** | 6.4929*** | 3.5938** | −3.5598 |
| | DOLS coefficient | 0.0449 | 0.1481 | 0.7083 | 0.3597 | −4.2850 |
| | DOLS t-stat | −5.0734*** | 3.0053** | 6.8796*** | 3.9732** | −3.7043 |
| Nigeria | ARDL coefficient | 0.1279 | 0.1038 | 0.5026 | 0.7725 | 6.1993 |
| | ARDL t-stat | 6.2917** | −2.4820** | 2.9184*** | 8.3974* | 4.0975 |
| | FMOLS coefficient | 0.1592 | 0.1384 | 0.5595 | 0.7594 | 5.9987 |
| | FMOLS t-stat | 5.8921** | −2.7891** | 2.8493*** | 7.9029* | 4.1296 |
| | DOLS coefficient | 0.1357 | 0.1198 | 0.5102 | 0.7628 | 6.0042 |
| | DOLS t-stat | 6.1183** | −2.4037** | 2.9575*** | 8.3290* | 4.1119 |
| Pakistan | ARDL coefficient | 0.5826 | 0.3827 | −0.3712 | 0.9428 | 5.1987 |
| | ARDL t-stat | 3.5938*** | 3.6826*** | 5.2877** | 7.2973* | 4.1907 |
| | FMOLS coefficient | 0.5492 | 0.4024 | −0.4582 | 0.7693 | 4.7288 |

| | | | | | | |
|---|---|---|---|---|---|---|
| | FMOLS t-stat | 3.0597*** | 3.6792*** | 3.0900** | 6.8298* | 4.5929 |
| | DOLS coefficient | 0.5599 | 0.3825 | −0.3540 | 0.9020 | 5.0005 |
| | DOLS t-stat | 3.6687*** | 3.6822*** | 5.4991** | 7.0664* | 4.2984 |
| Philippines | ARDL coefficient | 0.9858 | 0.4828 | 0.1123 | 0.1147 | −7.3875 |
| | ARDL t-stat | 1.5932* | −1.3922** | 1.5662* | 2.4826** | −3.4877 |
| | FMOLS coefficient | 0.9029 | 0.4723 | 0.1320 | 0.1418 | −7.0983 |
| | FMOLS t-stat | 1.6992* | −1.9095** | 1.9247* | 2.3019** | −3.0664 |
| | DOLS coefficient | 0.9576 | 0.4774 | 0.1219 | 0.1295 | −7.4929 |
| | DOLS t-stat | 1.5983* | −1.3791** | 1.5780* | 2.4595** | −3.4917 |
| Tunisia | ARDL coefficient | 0.0424 | 0.9382 | 0.2058 | 0.1785 | 3.4906 |
| | ARDL t-stat | −3.9586** | 2.4928** | 2.9846** | 3.2977*** | 3.0957 |
| | FMOLS coefficient | 0.0673 | 0.9522 | 0.2391 | 0.1892 | 3.9825 |
| | FMOLS t-stat | −3.0010** | 2.7907** | 2.8299** | 3.0090*** | 3.5050 |
| | DOLS coefficient | 0.0552 | 0.9380 | 0.2055 | 0.1761 | 3.4789 |
| | DOLS t-stat | −3.5829** | 2.4926** | 2.9847** | 3.2960*** | 3.0888 |
| Vietnam | ARDL coefficient | −0.2975 | 0.0192 | 0.3916 | 0.2988 | −5.0700 |
| | ARDL t-stat | 4.7003*** | 4.4987*** | −2.3815** | 1.5927** | −1.9875 |
| | FMOLS coefficient | −0.4028 | 0.0478 | 0.5919 | 0.3288 | −5.0082 |
| | FMOLS t-stat | 3.0981** | 3.0996*** | −2.3985** | 1.7720** | 1.8445 |
| | DOLS coefficient | −0.3045 | 0.0156 | 0.4176 | 0.3019 | −5.1998 |
| | DOLS t-stat | 4.7114*** | 4.4942*** | −2.3993** | 1.6728** | −1.7193 |
| Zambia | ARDL coefficient | −0.0052 | 0.5020 | 0.7122 | −0.4826 | 3.9608 |
| | ARDL t-stat | 1.5698*** | 5.2984* | 3.6836** | −7.2815* | 2.9996 |
| | FMOLS coefficient | −0.0568 | 0.5992 | 0.6988 | −0.5074 | 3.7902 |
| | FMOLS t-stat | 2.9996** | 4.9900* | 3.6093** | −5.9827* | 2.9696 |
| | DOLS coefficient | −0.0045 | 0.5446 | 0.7309 | −0.4988 | 3.9802 |
| | DOLS t-stat | 1.5788*** | 5.3929* | 3.8934** | −6.4999* | 2.9007 |

$X_1$ = number of confirmed COVID-19 cases; $X_2$ = balance of food trade; $X_3$ = food inflation; $X_4$ = currency exchange; *,**,*** = significance at 10% level, 5% level, and 1% level, respectively. Source: authors' development.

**Table S9.** FMOLS and DOLS tests results and ARDL long-run estimates, Group 3.

| Country | Parameters | $X_1$ | $X_2$ | $X_3$ | $X_4$ | Constant |
|---|---|---|---|---|---|---|
| Algeria | ARDL coefficient | 0.0486 | 0.7294 | −0.9621 | 0.1833 | −2.4985 |
| | ARDL t-stat | 2.6893* | 1.0487** | −1.2947* | −1.3028** | −2.0012 |
| | FMOLS coefficient | 0.0688 | 0.7045 | −0.9404 | 0.2291 | −2.4056 |
| | FMOLS t-stat | 2.4982* | 1.2928** | −1.3300* | −1.3098** | −2.0667 |
| | DOLS coefficient | 0.0506 | 0.7377 | −0.9501 | 0.1952 | −2.5029 |
| | DOLS t-stat | 2.6944* | 1.0483** | −1.2892* | −1.3111** | −2.0482 |
| Botswana | ARDL coefficient | −0.3985 | 0.4937 | 0.0385 | 0.3792 | 6.2875 |
| | ARDL t-stat | 4.2970* | −1.9473*** | 2.6973** | −1.9582** | 4.2099 |
| | FMOLS coefficient | −0.4892 | 0.5044 | 0.0928 | 0.4081 | 6.1103 |
| | FMOLS t-stat | 4.3929* | −1.8029*** | 2.5504** | −1.9772** | 4.2468 |
| | DOLS coefficient | −0.4078 | 0.4985 | 0.0682 | 0.3694 | 6.2901 |
| | DOLS t-stat | 4.2444* | −1.9471*** | 2.7010** | −1.9773** | 4.2399 |
| Colombia | ARDL coefficient | 0.0284 | 0.3309 | 0.1566 | 0.0462 | 4.1984 |
| | ARDL t-stat | 2.5897*** | 2.5824*** | 3.0521** | 2.3975*** | 3.0019 |
| | FMOLS coefficient | 0.0344 | 0.4090 | 0.1390 | 0.0628 | 4.1197 |
| | FMOLS t-stat | 2.5883*** | 2.9688*** | 3.1178** | 2.4199*** | 3.0666 |
| | DOLS coefficient | 0.0267 | 0.3486 | 0.1691 | 0.0591 | 4.2287 |
| | DOLS t-stat | 2.5890*** | 2.5852*** | 3.0664** | 2.4087*** | 3.1129 |
| Dominican Republic | ARDL coefficient | 0.0931 | 0.3892 | −0.1649 | 0.1284 | −5.3201 |
| | ARDL t-stat | 3.5863*** | 3.2989*** | 2.0593*** | 3.9701*** | −4.4865 |
| | FMOLS coefficient | 0.0882 | 0.3563 | −0.1550 | 0.1592 | −5.0021 |
| | FMOLS t-stat | 3.4456*** | 3.0674*** | 2.1675*** | 3.5826*** | −4.2354 |
| | DOLS coefficient | 0.0970 | 0.3797 | −0.1773 | 0.1381 | −5.3378 |
| | DOLS t-stat | 3.5868*** | 3.2593*** | 2.0646*** | 3.9882*** | −4.4997 |
| Ecuador | ARDL coefficient | 0.3987 | 0.1237 | 0.2900 | 0.5109 | 2.1995 |
| | ARDL t-stat | 9.3866*** | 7.2000** | −3.0482*** | −2.4975** | 1.8202 |
| | FMOLS coefficient | 0.4285 | 0.1492 | 0.3031 | 0.5582 | 2.3049 |
| | FMOLS t-stat | 8.0590*** | 7.0693** | −2.9997*** | −2.5699** | 1.9882 |
| | DOLS coefficient | 0.4122 | 0.1320 | 0.2906 | 0.5287 | 2.2003 |
| | DOLS t-stat | 9.0337*** | 7.3418** | −3.0485*** | −2.5086** | 1.8347 |
| Guatemala | ARDL coefficient | 0.1894 | 0.1748 | −0.3982 | 0.0473 | 4.9687 |
| | ARDL t-stat | 5.9027** | 5.3285** | −1.9467* | 4.2999* | 3.9198 |
| | FMOLS coefficient | 0.2291 | 0.2390 | −0.5029 | 0.0727 | 4.7605 |
| | FMOLS t-stat | 5.5003** | 5.0492** | −1.7895* | 4.0052* | 3.5977 |
| | DOLS coefficient | 0.1929 | 0.1866 | −0.4006 | 0.0591 | 4.7992 |
| | DOLS t-stat | 5.9167** | 5.3263** | −1.9530* | 4.3087* | 3.8845 |
| Iran | ARDL coefficient | −0.0480 | −0.4923 | −0.4888 | −0.0921 | 8.2977 |
| | ARDL t-stat | 3.0875* | 1.0480* | −7.0024** | 3.9612* | 5.2831 |
| | FMOLS coefficient | −0.0445 | −0.4994 | −0.5690 | −0.0941 | 8.1167 |
| | FMOLS t-stat | 2.9963* | 1.1126* | −6.8215** | 3.8093* | 5.2819 |
| | DOLS coefficient | −0.0592 | −0.4829 | −0.4874 | −0.0884 | 8.0034 |
| | DOLS t-stat | 3.0994* | 1.0558* | −7.0020** | 3.9519* | 5.0582 |
| Iraq | ARDL coefficient | 0.0378 | 0.0372 | −0.9387 | 0.1029 | −1.3087 |
| | ARDL t-stat | −5.9076** | 3.5559** | −3.0298* | 1.9444** | −1.0022 |
| | FMOLS coefficient | 0.0492 | 0.0599 | −0.8057 | 0.1393 | −1.4029 |
| | FMOLS t-stat | −5.5020** | 3.2918** | −3.2801* | 2.0001** | −1.1855 |
| | DOLS coefficient | 0.0420 | 0.0443 | −0.9491 | 0.1149 | −1.3192 |
| | DOLS t-stat | −5.9147** | 3.5692** | −3.1194* | 1.9467** | −1.1793 |
| Jordan | ARDL coefficient | −0.0663 | 0.9275 | 0.0019 | 0.4991 | −3.9866 |
| | ARDL t-stat | 4.5197* | 5.2985*** | 4.6972** | −7.2974*** | −2.3865 |
| | FMOLS coefficient | −0.0672 | 0.9426 | 0.0665 | 0.5591 | −3.0187 |
| | FMOLS t-stat | 4.3091* | 5.4029*** | 4.6722** | −7.1197*** | −2.7023 |
| | DOLS coefficient | −0.0554 | 0.9392 | 0.0391 | 0.5072 | −3.7724 |
| | DOLS t-stat | 4.5219* | 5.3026*** | 4.7084** | −7.0053*** | −2.3502 |
| Lebanon | ARDL coefficient | 0.1865 | 0.7291 | 0.4195 | 0.5190 | −6.2971 |
| | ARDL t-stat | 3.9777** | 3.5937*** | 5.0021** | −5.2741*** | −5.1989 |
| | FMOLS coefficient | 0.2038 | 0.7501 | 0.4928 | 0.5974 | −6.0029 |
| | FMOLS t-stat | 3.5026** | 3.3929*** | 5.0929** | −5.2986*** | −5.2988 |
| | DOLS coefficient | 0.1990 | 0.7167 | 0.4290 | 0.5228 | −6.3084 |
| | DOLS t-stat | 3.9963** | 3.5896*** | 5.0492** | −5.2894*** | −5.2097 |
| Libya | ARDL coefficient | −0.1993 | 0.7398 | 0.0417 | 0.6719 | 7.1082 |
| | ARDL t-stat | 4.0030** | −2.5697*** | 3.0585*** | 2.0034*** | 4.0884 |
| | FMOLS coefficient | −0.2096 | 0.7585 | 0.1583 | 0.5880 | 6.8201 |

| | | | | | | |
|---|---|---|---|---|---|---|
| | FMOLS t-stat | 4.1299** | −2.4406*** | 3.8771*** | 2.4389*** | 4.1004 |
| | DOLS coefficient | −0.1974 | 0.7492 | 0.1390 | 0.6756 | 7.0055 |
| | DOLS t-stat | 4.0502** | −2.5550*** | 3.0663*** | 2.0592*** | 4.0619 |
| Namibia | ARDL coefficient | −0.4975 | 0.2745 | 0.1116 | 0.3001 | −2.9853 |
| | ARDL t-stat | −3.0052* | 4.0032*** | −1.0470** | 4.2988*** | −1.0009 |
| | FMOLS coefficient | −0.5003 | 0.2890 | 0.1255 | 0.3184 | −2.7028 |
| | FMOLS t-stat | −2.9794* | 4.1199*** | −1.2837** | 4.0920*** | −1.0493 |
| | DOLS coefficient | −0.4840 | 0.2821 | 0.1289 | 0.3053 | −2.9685 |
| | DOLS t-stat | −3.0097* | 4.0173*** | −1.0571** | 4.2664*** | −1.0377 |
| Peru | ARDL coefficient | 0.5030 | 0.4982 | 0.4927 | 0.0287 | 4.9082 |
| | ARDL t-stat | 5.3977*** | 7.2412** | 3.0053* | −2.9695* | 1.0886 |
| | FMOLS coefficient | 0.5290 | 0.5945 | 0.5428 | 0.0333 | 4.5297 |
| | FMOLS t-stat | 5.4029** | 7.0031** | 3.1924* | −2.1876* | 1.2402 |
| | DOLS coefficient | 0.5116 | 0.4584 | 0.4499 | 0.0383 | 4.9105 |
| | DOLS t-stat | 5.3803** | 7.2559** | 3.0109* | −2.9555* | 1.0957 |
| Sri Lanka | ARDL coefficient | 0.2548 | −0.2655 | 0.5102 | −0.1859 | 6.0821 |
| | ARDL t-stat | −1.9642** | 1.5977* | 5.2260* | 9.4921** | 4.0982 |
| | FMOLS coefficient | 0.3384 | −0.3000 | 0.5358 | −0.1884 | 5.5085 |
| | FMOLS t-stat | −2.0985** | 1.6547* | 5.0485* | 8.4928** | 4.2256 |
| | DOLS coefficient | 0.2749 | −0.2694 | 0.5299 | −0.1940 | 5.7028 |
| | DOLS t-stat | −1.9774** | 1.5602* | 5.2346* | 9.5229** | 4.1993 |
| Turkey | ARDL coefficient | 0.0861 | 0.0826 | 0.3928 | 0.0182 | −5.9809 |
| | ARDL t-stat | 6.1864* | 4.2909* | −2.0532** | −2.1418*** | −4.9997 |
| | FMOLS coefficient | 0.0985 | 0.0662 | 0.4181 | 0.0382 | −5.0495 |
| | FMOLS t-stat | 5.9001 | 4.5093 | −2.1285** | −2.1867*** | −4.4009 |
| | DOLS coefficient | 0.0873 | 0.0739 | 0.4006 | 0.0286 | −5.8846 |
| | DOLS t-stat | 6.1965* | 4.3088* | −2.0693** | −2.1529*** | −4.8201 |

$X_1$ = number of confirmed COVID-19 cases; $X_2$ = balance of food trade; $X_3$ = food inflation; $X_4$ = currency exchange; *,**,*** = significance at 10% level, 5% level, and 1% level, respectively. Source: authors' development.

**Table S10.** TY causality test results, Group 1.

| Country | Parameters | Y→$X_1$ | $X_1$→Y | Y→$X_2$ | $X_2$→Y | Y→$X_3$ | $X_3$→Y | Y→$X_4$ | $X_4$→Y |
|---|---|---|---|---|---|---|---|---|---|
| Afghanistan | Test statistics | 0.39 | 1.94 | 2.09 | 9.48 | 0.55 | 0.18 | 2.64 | 3.19 |
| | *p* value | 0.49 | 0.33 | 0.31 | 0.06* | 0.47 | 0.62 | 0.26 | 0.20 |
| Burkina Faso | Test statistics | 5.40 | 9.16 | 4.69 | 5.10 | 0.97 | 2.53 | 8.18 | 27.05 |
| | *p* value | 0.31 | 0.18 | 0.41 | 0.33 | 0.84 | 0.52 | 0.23 | 0.06* |
| Chad | Test statistics | 12.37 | 4.47 | 6.01 | 9.13 | 8.05 | 21.04 | 0.96 | 2.11 |
| | *p* value | 0.14 | 0.33 | 0.26 | 0.19 | 0.22 | 0.00** | 0.77 | 0.42 |
| Congo, Democratic Republic | Test statistics | 5.32 | 1.11 | 7.15 | 4.04 | 3.18 | 17.28 | 8.14 | 10.43 |
| | *p* value | 0.28 | 0.75 | 0.16 | 0.35 | 0.40 | 0.00** | 0.13 | 0.07* |
| Ethiopia | Test statistics | 2.07 | 6.22 | 0.19 | 3.08 | 1.95 | 10.55 | 0.66 | 2.16 |
| | *p* value | 0.45 | 0.19 | 0.93 | 0.38 | 0.48 | 0.00*** | 0.79 | 0.43 |
| Guinea | Test statistics | 3.90 | 10.00 | 1.14 | 15.02 | 6.41 | 14.20 | 0.47 | 4.08 |
| | *p* value | 0.36 | 0.15 | 0.66 | 0.00** | 0.26 | 0.01* | 0.91 | 0.34 |
| Haiti | Test statistics | 2.28 | 3.11 | 1.05 | 8.39 | 2.02 | 4.13 | 1.59 | 0.72 |
| | *p* value | 0.35 | 0.23 | 0.56 | 0.00** | 0.38 | 0.17 | 0.41 | 0.77 |
| Mali | Test statistics | 2.18 | 11.63 | 0.44 | 1.17 | 0.16 | 5.14 | 0.98 | 7.00 |
| | *p* value | 0.13 | 0.00* | 0.41 | 0.20 | 0.92 | 0.06 | 0.23 | 0.03* |
| Mozambique | Test statistics | 0.30 | 3.01 | 2.12 | 6.54 | 0.14 | 4.13 | 0.56 | 1.19 |
| | *p* value | 0.72 | 0.23 | 0.30 | 0.02* | 0.91 | 0.15 | 0.61 | 0.47 |
| Nepal | Test statistics | 2.01 | 6.38 | 0.58 | 3.12 | 1.66 | 0.97 | 0.14 | 4.75 |
| | *p* value | 0.18 | 0.03* | 0.69 | 0.10 | 0.25 | 0.41 | 0.90 | 0.07* |
| Niger | Test statistics | 1.05 | 17.20 | 1.75 | 5.06 | 0.58 | 2.10 | 0.22 | 9.12 |
| | *p* value | 0.53 | 0.00** | 0.39 | 0.15 | 0.67 | 0.34 | 0.88 | 0.06* |
| Sierra Leone | Test statistics | 0.80 | 6.64 | 4.04 | 11.17 | 0.44 | 14.29 | 1.07 | 2.63 |
| | *p* value | 0.84 | 0.11 | 0.20 | 0.03* | 0.92 | 0.01** | 0.75 | 0.34 |
| Tajikistan | Test statistics | 1.29 | 3.13 | 0.18 | 16.20 | 1.03 | 13.84 | 0.53 | 8.66 |
| | *p* value | 0.45 | 0.21 | 0.82 | 0.00** | 0.48 | 0.05** | 0.76 | 0.08* |
| Tanzania | Test statistics | 7.17 | 20.40 | 0.81 | 4.05 | 2.19 | 37.08 | 0.44 | 1.07 |
| | *p* value | 0.22 | 0.09 | 0.75 | 0.34 | 0.55 | 0.00** | 0.83 | 0.71 |
| Yemen | Test statistics | 1.00 | 13.17 | 0.08 | 8.16 | 1.98 | 4.13 | 0.35 | 0.86 |
| | *p* value | 0.53 | 0.00* | 0.94 | 0.02** | 0.27 | 0.11 | 0.76 | 0.59 |

*Y* = number of people with insufficient food consumption; $X_1$ = number of confirmed COVID-19 cases; $X_2$ = balance of food trade; $X_3$ = food inflation; $X_4$ = currency exchange; *,**,*** = significance at 10% level, 5% level, and 1% level, respectively. Source: authors' development.

**Table S11.** TY causality test results, Group 2.

| Country | Parameters | $Y \to X_1$ | $X_1 \to Y$ | $Y \to X_2$ | $X_2 \to Y$ | $Y \to X_3$ | $X_3 \to Y$ | $Y \to X_4$ | $X_4 \to Y$ |
|---|---|---|---|---|---|---|---|---|---|
| Bangladesh | Test statistics | 0.68 | 8.13 | 0.99 | 4.36 | 0.14 | 2.01 | 0.05 | 1.16 |
| | *p* value | 0.41 | 0.00** | 0.30 | 0.07 | 0.70 | 0.18 | 0.96 | 0.27 |
| Bolivia | Test statistics | 1.16 | 7.07 | 0.75 | 4.19 | 2.03 | 8.55 | 0.10 | 0.17 |
| | *p* value | 0.33 | 0.02* | 0.47 | 0.11 | 0.25 | 0.00* | 0.84 | 0.80 |
| Cambodia | Test statistics | 0.20 | 3.00 | 0.28 | 2.65 | 1.04 | 6.14 | 0.55 | 0.96 |
| | *p* value | 0.77 | 0.08 | 0.71 | 0.14 | 0.32 | 0.00* | 0.46 | 0.37 |
| Cameroon | Test statistics | 0.52 | 5.15 | 0.33 | 13.07 | 0.96 | 3.02 | 0.12 | 1.44 |
| | *p* value | 0.49 | 0.11 | 0.58 | 0.00** | 0.35 | 0.17 | 0.79 | 0.26 |
| Cote d’Ivoire | Test statistics | 0.13 | 4.10 | 1.19 | 2.75 | 0.41 | 12.43 | 1.02 | 7.22 |
| | *p* value | 0.90 | 0.08 | 0.30 | 0.21 | 0.76 | 0.00** | 0.34 | 0.01* |
| India | Test statistics | 0.37 | 23.15 | 0.55 | 6.12 | 1.12 | 2.08 | 1.76 | 15.06 |
| | *p* value | 0.83 | 0.00*** | 0.79 | 0.13 | 0.47 | 0.32 | 0.39 | 0.01* |
| Indonesia | Test statistics | 0.85 | 10.28 | 1.00 | 1.29 | 0.22 | 7.36 | 0.04 | 5.15 |
| | *p* value | 0.31 | 0.00*** | 0.23 | 0.16 | 0.65 | 0.01* | 0.90 | 0.02* |
| Kenya | Test statistics | 0.67 | 6.11 | 0.24 | 6.88 | 1.02 | 6.00 | 1.31 | 2.03 |
| | *p* value | 0.46 | 0.01** | 0.72 | 0.00** | 0.29 | 0.02* | 0.24 | 0.11 |
| Kyrgyzstan | Test statistics | 1.13 | 2.05 | 1.60 | 7.17 | 0.53 | 8.23 | 1.02 | 0.80 |
| | *p* value | 0.34 | 0.12 | 0.23 | 0.00** | 0.65 | 0.00** | 0.37 | 0.41 |
| Nigeria | Test statistics | 0.15 | 9.70 | 0.41 | 3.85 | 1.40 | 10.05 | 0.06 | 0.97 |
| | *p* value | 0.77 | 0.02** | 0.56 | 0.10 | 0.22 | 0.00** | 0.81 | 0.29 |
| Pakistan | Test statistics | 0.08 | 3.19 | 0.20 | 1.07 | 0.85 | 6.13 | 0.48 | 14.63 |
| | *p* value | 0.92 | 0.25 | 0.81 | 0.42 | 0.49 | 0.14 | 0.66 | 0.00** |
| Philippines | Test statistics | 0.89 | 4.24 | 0.74 | 16.30 | 1.15 | 10.47 | 0.33 | 2.01 |
| | *p* value | 0.47 | 0.14 | 0.55 | 0.01*** | 0.42 | 0.03** | 0.78 | 0.30 |
| Tunisia | Test statistics | 0.18 | 3.03 | 0.26 | 12.98 | 0.09 | 9.13 | 0.60 | 1.24 |
| | *p* value | 0.72 | 0.11 | 0.67 | 0.00*** | 0.90 | 0.01** | 0.51 | 0.25 |
| Vietnam | Test statistics | 0.44 | 0.70 | 0.02 | 2.01 | 0.05 | 5.65 | 0.14 | 1.18 |
| | *p* value | 0.56 | 0.34 | 0.95 | 0.13 | 0.86 | 0.00** | 0.77 | 0.22 |
| Zambia | Test statistics | 0.33 | 0.52 | 0.21 | 3.30 | 0.97 | 7.04 | 0.09 | 1.24 |
| | *p* value | 0.65 | 0.57 | 0.74 | 0.14 | 0.41 | 0.01** | 0.88 | 0.30 |

$Y$ = number of people with insufficient food consumption; $X_1$ = number of confirmed COVID-19 cases; $X_2$ = balance of food trade; $X_3$ = food inflation; $X_4$ = currency exchange; *,**,*** = significance at 10% level, 5% level, and 1% level, respectively. Source: authors’ development.

**Table S12.** TY causality test results, Group 3.

| Country | Parameters | $Y \to X_1$ | $X_1 \to Y$ | $Y \to X_2$ | $X_2 \to Y$ | $Y \to X_3$ | $X_3 \to Y$ | $Y \to X_4$ | $X_4 \to Y$ |
|---|---|---|---|---|---|---|---|---|---|
| Algeria | Test statistics | 0.44 | 12.01 | 0.98 | 10.43 | 1.65 | 4.04 | 0.12 | 2.77 |
| | *p* value | 0.56 | 0.00** | 0.37 | 0.01*** | 0.29 | 0.11 | 0.87 | 0.20 |
| Botswana | Test statistics | 0.20 | 3.70 | 0.49 | 13.67 | 0.94 | 1.99 | 1.05 | 9.14 |
| | *p* value | 0.77 | 0.13 | 0.58 | 0.00*** | 0.39 | 0.24 | 0.36 | 0.01*** |
| Colombia | Test statistics | 0.73 | 32.18 | 4.01 | 25.40 | 2.11 | 9.13 | 1.40 | 19.46 |
| | *p* value | 0.81 | 0.00*** | 0.24 | 0.01*** | 0.38 | 0.10 | 0.66 | 0.02*** |
| Dominican Republic | Test statistics | 0.04 | 9.74 | 0.72 | 2.18 | 0.13 | 1.06 | 0.85 | 7.03 |
| | *p* value | 0.92 | 0.00** | 0.34 | 0.11 | 0.75 | 0.23 | 0.29 | 0.01*** |
| Ecuador | Test statistics | 0.85 | 19.13 | 0.31 | 5.25 | 1.80 | 11.20 | 1.03 | 2.17 |
| | *p* value | 0.46 | 0.00*** | 0.69 | 0.12 | 0.31 | 0.02* | 0.40 | 0.25 |
| Guatemala | Test statistics | 0.12 | 0.95 | 0.44 | 7.14 | 1.03 | 2.19 | 0.21 | 1.44 |
| | *p* value | 0.85 | 0.34 | 0.47 | 0.00** | 0.31 | 0.15 | 0.66 | 0.26 |
| Iran | Test statistics | 0.24 | 12.07 | 0.85 | 6.25 | 0.40 | 1.02 | 1.55 | 2.07 |
| | *p* value | 0.81 | 0.00** | 0.56 | 0.13 | 0.72 | 0.42 | 0.36 | 0.30 |
| Iraq | Test statistics | 0.09 | 1.66 | 1.05 | 5.80 | 0.19 | 0.81 | 0.38 | 0.62 |
| | *p* value | 0.83 | 0.18 | 0.23 | 0.02** | 0.65 | 0.28 | 0.47 | 0.34 |
| Jordan | Test statistics | 1.04 | 4.55 | 0.88 | 18.25 | 1.38 | 12.46 | 0.20 | 2.17 |
| | *p* value | 0.37 | 0.12 | 0.42 | 0.00*** | 0.30 | 0.01* | 0.79 | 0.21 |
| Lebanon | Test statistics | 0.06 | 1.36 | 0.99 | 8.14 | 0.81 | 6.09 | 0.14 | 0.43 |
| | *p* value | 0.92 | 0.14 | 0.25 | 0.00*** | 0.28 | 0.01* | 0.75 | 0.51 |
| Libya | Test statistics | 0.11 | 3.03 | 0.30 | 10.02 | 1.40 | 1.83 | 0.62 | 0.80 |
| | *p* value | 0.84 | 0.10 | 0.64 | 0.01*** | 0.24 | 0.19 | 0.49 | 0.45 |
| Namibia | Test statistics | 0.39 | 0.67 | 1.21 | 9.27 | 0.97 | 7.23 | 0.81 | 6.08 |
| | *p* value | 0.58 | 0.41 | 0.13 | 0.00*** | 0.25 | 0.01* | 0.34 | 0.02*** |
| Peru | Test statistics | 0.17 | 14.19 | 0.50 | 10.34 | 1.04 | 2.15 | 0.77 | 8.40 |
| | *p* value | 0.72 | 0.00*** | 0.55 | 0.01* | 0.27 | 0.13 | 0.39 | 0.02*** |
| Sri Lanka | Test statistics | 0.15 | 9.08 | 0.32 | 1.13 | 0.93 | 5.12 | 0.55 | 4.00 |
| | *p* value | 0.80 | 0.00*** | 0.67 | 0.15 | 0.21 | 0.01* | 0.48 | 0.02*** |
| Turkey | Test statistics | 0.28 | 15.06 | 0.44 | 2.17 | 1.55 | 10.41 | 0.96 | 9.35 |
| | *p* value | 0.74 | 0.00*** | 0.58 | 0.16 | 0.24 | 0.01** | 0.31 | 0.02*** |

$Y$ = number of people with insufficient food consumption; $X_1$ = number of confirmed COVID-19 cases; $X_2$ = balance of food trade; $X_3$ = food inflation; $X_4$ = currency exchange; *,**,*** = significance at 10% level, 5% level, and 1% level, respectively. Source: authors' development.

**Table S13.** Variance decomposition of *Y* over a nine periods (three quarters) horizon, Group 1.

| Country | Period | Standard error | Y | $X_1$ | $X_2$ | $X_3$ | $X_4$ |
|---|---|---|---|---|---|---|---|
| Afghanistan | September 2020 | 0.004726 | 93.178533 | 0.310963 | 4.889038 | 0.012871 | 1.608595 |
| | December 2020 | 0.007688 | 88.899731 | 1.902381 | 5.904815 | 0.009842 | 3.283231 |
| | March 2021 | 0.012864 | 83.982772 | 2.159286 | 8.007200 | 0.008275 | 5.842467 |
| Burkina Faso | September 2020 | 0.003719 | 95.829400 | 0.499841 | 0.994106 | 0.031578 | 2.645075 |
| | December 2020 | 0.005851 | 89.492855 | 3.159844 | 0.752981 | 0.149811 | 6.444509 |
| | March 2021 | 0.010509 | 84.203371 | 5.809420 | 0.707732 | 0.266082 | 9.013395 |
| Chad | September 2020 | 0.006187 | 92.178925 | 0.586849 | 1.568395 | 5.498254 | 0.167577 |
| | December 2020 | 0.008468 | 82.400067 | 2.377412 | 4.189734 | 10.309615 | 0.723172 |
| | March 2021 | 0.011921 | 76.398555 | 3.096329 | 5.095246 | 13.704832 | 1.705038 |
| Congo, Democratic Republic | September 2020 | 0.007164 | 94.117593 | 0.042881 | 0.808432 | 3.177245 | 1.853849 |
| | December 2020 | 0.013877 | 90.058290 | 0.061299 | 0.504914 | 5.909843 | 3.465654 |
| | March 2021 | 0.021955 | 81.174509 | 0.078531 | 0.439886 | 11.120942 | 7.186132 |
| Ethiopia | September 2020 | 0.010297 | 93.148096 | 1.309442 | 0.320833 | 5.100985 | 0.120644 |
| | December 2020 | 0.014958 | 85.109844 | 1.580413 | 1.225821 | 10.579216 | 1.504706 |
| | March 2021 | 0.021870 | 77.739052 | 1.690286 | 3.000632 | 14.840523 | 2.729507 |
| Guinea | September 2020 | 0.009421 | 92.203361 | 1.009850 | 3.172805 | 2.849863 | 0.764121 |
| | December 2020 | 0.015924 | 84.640840 | 1.202587 | 7.404022 | 6.403445 | 0.349106 |
| | March 2021 | 0.020071 | 73.129065 | 2.009524 | 12.511563 | 11.505621 | 0.844227 |
| Haiti | September 2020 | 0.008419 | 95.148984 | 0.704533 | 3.405712 | 0.498423 | 0.242348 |
| | December 2020 | 0.013957 | 88.350902 | 2.567071 | 6.139209 | 1.309884 | 1.632934 |
| | March 2021 | 0.017299 | 80.772309 | 3.120067 | 11.042261 | 2.522567 | 2.542796 |
| Mali | September 2020 | 0.028555 | 94.909951 | 2.084932 | 0.042220 | 0.438975 | 2.523922 |
| | December 2020 | 0.034800 | 89.048255 | 5.156625 | 0.068285 | 0.707532 | 5.019303 |
| | March 2021 | 0.041985 | 82.455084 | 9.126083 | 0.099321 | 0.598340 | 7.721172 |
| Mozambique | September 2020 | 0.005680 | 98.028851 | 0.199874 | 1.117972 | 0.407162 | 0.246141 |
| | December 2020 | 0.007996 | 96.538923 | 0.219085 | 1.248065 | 0.289875 | 1.704052 |
| | March 2021 | 0.010077 | 93.119874 | 0.259874 | 2.802876 | 0.209876 | 3.607500 |
| Nepal | September 2020 | 0.037945 | 94.894071 | 3.170932 | 0.289403 | 0.049841 | 1.595753 |
| | December 2020 | 0.043886 | 87.095286 | 6.809884 | 0.309724 | 0.059875 | 5.725231 |
| | March 2021 | 0.052893 | 79.479210 | 10.290955 | 0.552986 | 0.069951 | 9.606898 |
| Niger | September 2020 | 0.004526 | 95.001758 | 2.502111 | 0.904713 | 0.147804 | 1.443614 |
| | December 2020 | 0.008270 | 88.358921 | 6.142203 | 1.005892 | 0.170987 | 4.321997 |
| | March 2021 | 0.013877 | 82.086743 | 9.459286 | 1.580396 | 0.225803 | 6.647772 |
| Sierra Leone | September 2020 | 0.009884 | 96.400027 | 0.189775 | 1.009974 | 2.323875 | 0.076349 |
| | December 2020 | 0.016872 | 93.116405 | 0.540096 | 1.130843 | 4.950972 | 0.261684 |
| | March 2021 | 0.027881 | 90.400933 | 0.833309 | 1.308875 | 6.912865 | 0.544018 |
| Tajikistan | September 2020 | 0.001873 | 92.415807 | 0.084925 | 4.058277 | 2.140723 | 1.300268 |
| | December 2020 | 0.004920 | 85.190287 | 0.113067 | 9.409287 | 4.086287 | 1.201072 |
| | March 2021 | 0.008344 | 77.097630 | 0.140992 | 14.006000 | 7.528751 | 1.226627 |
| Tanzania | September 2020 | 0.003835 | 95.230875 | 0.905298 | 0.428515 | 3.258826 | 0.176486 |
| | December 2020 | 0.008006 | 91.360922 | 1.230954 | 0.666192 | 6.003710 | 0.738222 |
| | March 2021 | 0.013774 | 87.110943 | 1.503985 | 1.003985 | 9.294986 | 1.086101 |
| Yemen | September 2020 | 0.004217 | 96.370849 | 2.029486 | 1.398412 | 0.197746 | 0.003507 |
| | December 2020 | 0.007399 | 93.029871 | 5.391854 | 1.209864 | 0.308592 | 0.059819 |
| | March 2021 | 0.013985 | 89.427849 | 8.298401 | 1.332875 | 0.808521 | 0.132354 |

*Y* = number of people with insufficient food consumption; $X_1$ = number of confirmed COVID-19 cases; $X_2$ = balance of food trade; $X_3$ = food inflation; $X_4$ = currency exchange. Source: authors' development

**Table S14.** Variance decomposition of *Y* over a nine periods (three quarters) horizon, Group 2.

| Country | Period | Standard error | Y | $X_1$ | $X_2$ | $X_3$ | $X_4$ |
|---|---|---|---|---|---|---|---|
| Bangladesh | September 2020 | 0.007390 | 95.370145 | 3.038921 | 0.835521 | 0.405194 | 0.350219 |
| | December 2020 | 0.009186 | 88.409283 | 8.309127 | 1.492300 | 0.951096 | 0.838194 |
| | March 2021 | 0.013909 | 82.559531 | 12.095140 | 2.649302 | 1.305821 | 1.390206 |
| Bolivia | September 2020 | 0.004008 | 93.094854 | 2.179285 | 0.304875 | 4.382022 | 0.038964 |
| | December 2020 | 0.006927 | 85.104820 | 4.539953 | 1.707328 | 8.626945 | 0.020954 |
| | March 2021 | 0.012815 | 79.372041 | 4.629850 | 1.920584 | 14.059184 | 0.018341 |
| Cambodia | September 2020 | 0.008510 | 94.072918 | 0.850211 | 0.670913 | 4.297705 | 0.108253 |
| | December 2020 | 0.011904 | 86.349271 | 1.590932 | 1.298584 | 10.110627 | 0.650586 |
| | March 2021 | 0.015991 | 80.230950 | 1.904863 | 1.840276 | 15.270843 | 0.753068 |
| Cameroon | September 2020 | 0.004896 | 96.398705 | 0.492125 | 2.509110 | 0.402782 | 0.197278 |
| | December 2020 | 0.008733 | 94.180437 | 0.908814 | 3.622819 | 0.763986 | 0.523944 |
| | March 2021 | 0.013005 | 91.309665 | 1.205985 | 5.389833 | 1.110321 | 0.984196 |
| Cote d'Ivoire | September 2020 | 0.011437 | 92.148064 | 0.039844 | 0.001965 | 4.297180 | 3.512947 |
| | December 2020 | 0.015042 | 85.002978 | 0.088301 | 0.002600 | 7.603291 | 7.302830 |
| | March 2021 | 0.020046 | 77.169285 | 0.105932 | 0.005928 | 12.437220 | 10.281635 |
| India | September 2020 | 0.006281 | 93.048772 | 3.793780 | 0.084725 | 0.005921 | 3.066802 |
| | December 2020 | 0.009942 | 80.279901 | 10.841954 | 0.009486 | 0.003912 | 8.864747 |
| | March 2021 | 0.014244 | 74.194296 | 14.498776 | 0.003910 | 0.002030 | 11.300988 |
| Indonesia | September 2020 | 0.005530 | 95.117295 | 1.903875 | 0.329855 | 1.527541 | 1.121434 |
| | December 2020 | 0.008408 | 90.930758 | 3.010410 | 0.450849 | 2.819885 | 2.788098 |
| | March 2021 | 0.010727 | 84.503985 | 5.529514 | 0.662976 | 5.028401 | 4.275124 |
| Kenya | September 2020 | 0.013166 | 92.004956 | 2.270498 | 3.427501 | 2.004976 | 0.292069 |
| | December 2020 | 0.020574 | 85.384109 | 4.389875 | 6.840035 | 2.883090 | 0.502891 |
| | March 2021 | 0.028218 | 79.403770 | 6.147930 | 10.131840 | 3.620996 | 0.695464 |
| Kyrgyzstan | September 2020 | 0.031096 | 93.920471 | 0.505149 | 2.820951 | 2.709857 | 0.043572 |
| | December 2020 | 0.049194 | 86.079288 | 1.301942 | 6.662188 | 5.908173 | 0.048409 |
| | March 2021 | 0.061702 | 78.222960 | 1.839585 | 10.909382 | 8.969566 | 0.058507 |
| Nigeria | September 2020 | 0.010025 | 97.481042 | 0.707928 | 0.392665 | 1.336629 | 0.081736 |
| | December 2020 | 0.015036 | 94.120953 | 1.259744 | 0.780482 | 3.703420 | 0.135401 |
| | March 2021 | 0.020738 | 90.893655 | 1.936021 | 0.928777 | 6.059754 | 0.181793 |
| Pakistan | September 2020 | 0.002915 | 96.529501 | 0.078375 | 0.038860 | 0.250285 | 3.102979 |
| | December 2020 | 0.004172 | 91.692750 | 0.050270 | 0.025827 | 0.508822 | 7.722331 |
| | March 2021 | 0.005986 | 87.069273 | 0.046206 | 0.022041 | 0.936606 | 11.925874 |
| Philippines | September 2020 | 0.011503 | 95.850002 | 0.102985 | 2.739824 | 1.269412 | 0.037777 |
| | December 2020 | 0.014837 | 89.104877 | 0.260387 | 5.853920 | 4.503865 | 0.276951 |
| | March 2021 | 0.019545 | 81.329586 | 0.449286 | 9.845090 | 7.971921 | 0.404117 |
| Tunisia | September 2020 | 0.002694 | 96.027573 | 0.409219 | 1.960987 | 1.508863 | 0.093358 |
| | December 2020 | 0.004885 | 88.560498 | 0.912876 | 5.340061 | 4.592095 | 0.594470 |
| | March 2021 | 0.006603 | 82.324409 | 1.204828 | 8.328612 | 7.509287 | 0.632864 |
| Vietnam | September 2020 | 0.003047 | 94.238894 | 0.008021 | 0.803267 | 4.380486 | 0.569332 |
| | December 2020 | 0.003819 | 85.099286 | 0.010587 | 2.209864 | 11.220061 | 1.460202 |
| | March 2021 | 0.004500 | 78.836502 | 0.013976 | 3.390510 | 15.984744 | 1.774268 |
| Zambia | September 2020 | 0.012053 | 96.110253 | 0.009310 | 0.802976 | 2.797056 | 0.280405 |
| | December 2020 | 0.017048 | 90.492870 | 0.012096 | 1.740965 | 7.089942 | 0.664127 |
| | March 2021 | 0.022729 | 84.950397 | 0.023051 | 2.498600 | 11.598730 | 0.929222 |

$Y$ = number of people with insufficient food consumption; $X_1$ = number of confirmed COVID-19 cases; $X_2$ = balance of food trade; $X_3$ = food inflation; $X_4$ = currency exchange. Source: authors' development.

**Table S15.** Variance decomposition of *Y* over a nine periods (three quarters) horizon, Group 3.

| Country | Period | Standard error | Y | $X_1$ | $X_2$ | $X_3$ | $X_4$ |
|---|---|---|---|---|---|---|---|
| Algeria | September 2020 | 0.005105 | 93.194765 | 3.509961 | 3.029875 | 0.249287 | 0.016112 |
| | December 2020 | 0.006238 | 87.088402 | 5.439854 | 6.798271 | 0.602855 | 0.070618 |
| | March 2021 | 0.008429 | 80.398771 | 8.346723 | 10.459914 | 0.710592 | 0.084000 |
| Botswana | September 2020 | 0.011730 | 95.459287 | 0.030952 | 2.329860 | 0.004293 | 2.175608 |
| | December 2020 | 0.013096 | 88.500233 | 0.043976 | 5.549811 | 0.006610 | 5.899370 |
| | March 2021 | 0.015387 | 81.003520 | 0.060293 | 9.049824 | 0.007298 | 9.879065 |
| Colombia | September 2020 | 0.009473 | 92.984556 | 2.498662 | 2.285527 | 0.013853 | 2.217402 |
| | December 2020 | 0.015905 | 83.150851 | 6.339876 | 5.340298 | 0.039761 | 5.129214 |
| | March 2021 | 0.021376 | 75.425932 | 8.930194 | 7.770392 | 0.050030 | 7.823452 |
| Dominican Republic | September 2020 | 0.006604 | 96.660496 | 1.802844 | 0.009930 | 0.001976 | 1.524754 |
| | December 2020 | 0.011975 | 94.026822 | 3.005985 | 0.012850 | 0.027887 | 2.926456 |
| | March 2021 | 0.025400 | 92.339524 | 4.238772 | 0.016927 | 0.059364 | 3.345413 |
| Ecuador | September 2020 | 0.003167 | 95.782758 | 2.347703 | 0.035723 | 1.809863 | 0.023953 |
| | December 2020 | 0.007341 | 91.298700 | 4.063911 | 0.266540 | 3.987576 | 0.383273 |
| | March 2021 | 0.009938 | 86.600382 | 6.909257 | 0.547101 | 5.145527 | 0.797733 |
| Guatemala | September 2020 | 0.037076 | 96.212805 | 0.028660 | 2.598520 | 0.795872 | 0.364143 |
| | December 2020 | 0.058351 | 90.829811 | 0.050999 | 6.420056 | 1.903986 | 0.795148 |
| | March 2021 | 0.072956 | 85.628403 | 0.079375 | 9.739851 | 3.597704 | 0.954667 |
| Iran | September 2020 | 0.015632 | 94.000470 | 3.850294 | 1.847442 | 0.008209 | 0.293585 |
| | December 2020 | 0.021693 | 88.198755 | 8.442958 | 2.756914 | 0.012985 | 0.588388 |
| | March 2021 | 0.028407 | 82.410007 | 12.438750 | 4.204920 | 0.017666 | 0.928657 |
| Iraq | September 2020 | 0.003791 | 93.783922 | 0.108387 | 6.019842 | 0.079375 | 0.008474 |
| | December 2020 | 0.005839 | 84.837763 | 0.940842 | 13.639843 | 0.512097 | 0.069455 |
| | March 2021 | 0.007230 | 78.592855 | 2.375554 | 16.945677 | 1.604982 | 0.480932 |
| Jordan | September 2020 | 0.017266 | 95.676044 | 0.023985 | 2.294875 | 1.999503 | 0.005593 |
| | December 2020 | 0.023404 | 89.039486 | 0.049583 | 6.259286 | 4.639504 | 0.012141 |
| | March 2021 | 0.030487 | 83.928051 | 0.062975 | 9.502850 | 6.482753 | 0.023371 |
| Lebanon | September 2020 | 0.045509 | 97.456629 | 0.400398 | 1.179387 | 0.948655 | 0.014931 |
| | December 2020 | 0.059172 | 94.773892 | 0.689956 | 3.039866 | 1.399840 | 0.096446 |
| | March 2021 | 0.064096 | 91.054496 | 0.779287 | 6.249302 | 1.785744 | 0.131171 |
| Libya | September 2020 | 0.005303 | 93.111590 | 1.029870 | 5.309978 | 0.501153 | 0.047409 |
| | December 2020 | 0.006092 | 82.529857 | 3.703998 | 12.649664 | 0.992501 | 0.123980 |
| | March 2021 | 0.008839 | 75.395602 | 5.123094 | 18.028750 | 1.139622 | 0.312932 |
| Namibia | September 2020 | 0.015827 | 96.340698 | 0.004935 | 1.592873 | 1.200980 | 0.860514 |
| | December 2020 | 0.038094 | 85.288095 | 0.017948 | 6.660255 | 3.694053 | 4.339649 |
| | March 2021 | 0.057022 | 73.109873 | 0.038761 | 12.197768 | 6.329755 | 8.323843 |
| Peru | September 2020 | 0.002504 | 92.039890 | 4.299756 | 2.019635 | 0.018720 | 1.621999 |
| | December 2020 | 0.003777 | 80.208444 | 9.029847 | 4.130297 | 0.029453 | 6.601959 |
| | March 2021 | 0.004920 | 71.559215 | 13.294860 | 6.200018 | 0.049841 | 8.896066 |
| Sri Lanka | September 2020 | 0.004406 | 95.386875 | 1.950200 | 0.040941 | 1.597470 | 1.024514 |
| | December 2020 | 0.005003 | 92.454822 | 2.305974 | 0.062216 | 2.500146 | 2.676842 |
| | March 2021 | 0.005698 | 89.967249 | 3.260016 | 0.077295 | 2.809897 | 3.885543 |
| Turkey | September 2020 | 0.002906 | 90.479904 | 4.110062 | 0.029873 | 2.848211 | 2.531950 |
| | December 2020 | 0.003351 | 79.502073 | 7.019281 | 0.049286 | 3.198602 | 10.230758 |
| | March 2021 | 0.003855 | 68.327048 | 11.309555 | 0.060337 | 5.089936 | 15.213124 |

$Y$ = number of people with insufficient food consumption; $X_1$ = number of confirmed COVID-19 cases; $X_2$ = balance of food trade; $X_3$ = food inflation; $X_4$ = currency exchange. Source: authors' development.
